# The Influence of Cryopreservation and Low-Temperature Seed Storage on the Morphological and Agronomical Characteristics of Fiber Flax

**DOI:** 10.3390/plants15040602

**Published:** 2026-02-13

**Authors:** Andrey V. Pavlov, Elizaveta A. Porokhovinova, Aleksandr V. Pavlov, Irina V. Kiseleva, Nina B. Brutch

**Affiliations:** 1 Department of Oil and Fiber Crops, N.I. Vavilov All-Russian Institute of Plant Genetic Resources (VIR), 42–44 B. Morskaya Str., 190000 St. Petersburg, Russia; avpavlov77@yandex.ru (A.V.P.); n.brutch@vir.nw.ru (N.B.B.); 2 Laboratory of Long-Term Plants Genetic Resources Preservation, N.I. Vavilov All-Russian Institute of Plant Genetic Resources (VIR), 42–44 B. Morskaya Str., 190000 St. Petersburg, Russia; pavlov-al@bk.ru; 3 A.A. Smorodintsev Department of Virology and Immunology, Institute of Experimental Medicine (IEM), 12 Acad. Pavlov Str., 197022 St. Petersburg, Russia; irina.v.kiseleva@mail.ru

**Keywords:** *Linum usitatissimum*, low temperature storage, cryopreservation, GeneBank, long-term modification, seeds germination, vegetative period phases, morphological characters, plant productivity, fiber quality

## Abstract

For the development of effective and secure methods for plant genetic resources preservation, different storage treatments of fiber flax seeds were compared. Seeds of the flax variety Orshanskiy-2 in aluminum foil bags were stored at different low temperatures, including in liquid nitrogen. Agronomic characters of plants grown from them and next-generation seeds were compared. Plants grown from frozen seeds changed 14 out of 31 evaluated characters in comparison with the non-frozen control. The biggest changes were detected after gradual freezing in liquid nitrogen, due to mechanical damage of the seed coat, and storage at −10 °C for 24 years. Freezing had a negative effect on production characters (straw, fiber and seed) because of the reduction of the germinated plant number. Seeds stored for 24 years at −10 °C, compared to control plants, ripened earlier, grew higher, produced a greater yield of straw and fiber, but had reduced fiber quality and increased seed size. Plants of the next generation showed a tendency toward attenuation of the storage time influence on flax characters. However, it is unknown how many years this process will take. For seed preservation in GeneBanks, it is recommended to use several variants of storage conditions and use rapid cooling and/or cryoprotectors. The latter two methods, which have been successfully used for other crops, should be implemented only after preliminary experiments.

## 1. Introduction

Preservation and conservation of plant diversity, and mainly of agricultural crops, are the basis of food security, especially under changing climate conditions [1,2,3,4,5]. The best way of genetic diversity maintenance is the long-term storage of seeds. Each seed preserves the whole genome of the plant and forms functional plants without special technologies [6]. Some seeds may preserve their viability for a very long time [7,8,9,10], but seeds of different plants have different potential viability, which also depends on the storage conditions [11,12,13]. In the majority of cases, seeds need to be periodically reproduced. Development of seed collections of plant genetic resources leads to increasing expenses required for the collections’ maintenance and, on the other hand, to the risk of mechanical and biological contamination.

In that regard, seeds have been stored in permafrost for a long time. This is a cheap, natural, and ecological method. Also, seeds in such storage can survive natural and anthropogenic disasters [14,15,16]. Seed collections have already been preserved for a long time in a depleted coal mine in Yakutia [17]. Also, a special underground seed storage, “Svalbard Global Seed Vault,” was built on Svalbard Island [17].

The experiments carried out with different crops in different conditions showed that seed longevity could be significantly increased by their storage at low temperatures, ~−20 °C, and low moisture content [18,19,20,21]. During such experiments, cytological analyses [22] and seed viability monitoring were carried out. For flax, it was discovered that the period of safe storage (that is, death of only 10% of seeds) is 33 years. It was concluded that 50% of seeds will die after approximately 88 years in storage [23,24]. Perspectives of using low temperatures for storage of seeds from wild species with different forms of physiological dormancy were also evaluated. Seeds of 16 species in the stage of coercive and superficial dormancy preserved germination ability after 9 years of storage at −20 °C [25]. Some scientists also suggest the storage of seeds at low positive temperatures (+4 °C) as a compromise, a less expensive variant of storage [26].

Since the end of the 20th century, storage of biological material has started to translate into GeneBanks [27]. Official FAO data includes 1750 GeneBanks preserving about 7.4 million accessions [27,28,29,30]. The main aim of their foundation is the maintenance of agricultural crops’ biodiversity, which nowadays is starting to decline [31,32,33,34,35].

Currently, it has become evident that *ex situ* biodiversity conservation appears more successful then *in situ* conservation, as this method is approximately 100 times cheaper, and preserved material is easily available [36]. Also, further development of biotechnological techniques will lead to a reduction in the number of genotypes required for preservation [37]. The GeneBanks preserve a large number of gene alleles and their combinations resulting from long-term plant evolution. Currently, it is not possible to recreate them using genetic editing techniques. Therefore, GeneBanks collections will remain useful for determining gene functions [21,38,39]. At the same time, biotechnology and gene editing methods help utilize this information and improve the function of GeneBanks [40].

For successful long-term seed storage at low temperatures, effective methods to maintain viability are needed. Predicted germination ability often appears to be higher than actual values. At the same time, these calculations are rather approximate [3,41,42,43].

Currently, different groups of scientists are searching for effective methods of testing the viability of preserved plant material [44,45,46,47]. The development of new methods increases the effectiveness of seed viability testing [48,49,50,51,52,53]. Methods of molecular genetics assist in determining the degree of similarity between accessions and finding alleles promising for breeding [54], which also helps to avoid duplication [55].

Widespread sequencing of accessions stored in GeneBanks generates a lot of new data, and this creates opportunities for future research and development. Currently existing forms of biological diversity have evolved for millions of years, and they provide us with essential material for improving the productivity, sustainability, and nutritional quality of food, fiber, fuel, and other resources that are critical for human well-being [56].

The viability of stored seeds depends on their initial quality. For selection of the best material for long-term storage, different methods are used: spectroscopy [57], X-ray [58], express radicle emergence test [59], and different cytological tests [60].

At present, alongside low-temperature seed storage, cryopreservation is used for long-term storage of the most valuable seeds [61,62,63,64,65,66,67,68,69]. This method is used for several valuable agricultural crops: cereals [49]; carrot [70]; green bean [71]; coffee, vigna [72]; seeds of many fruit trees such as apricot, apple, pear [73,74,75,76,77,78]; oil crops including flax [79]; different wild species [80,81,82,83,84,85,86]; and citrus [87].

Seeds of different crops have many physiological differences. So, methods of conservation must be adapted to their specific features. In this regard, methods of using cryoprotectors for vitrification were developed. These techniques are successfully used for seeds of orchids [88,89], tropical plants, and different endemics [90,91,92,93,94,95,96,97,98,99]. Also, they are used for medicinal herbs [100,101,102,103,104,105,106,107].

At the same time, successful long-term storage of seeds requires not only the preservation of their germination ability, but also maintenance of their genetic and phenotypic identity. There are a few investigations devoted to this problem [49,108]. It was discovered that cryopreservation modified germination and seedlings’ growth, but did not change characteristics of adult sorghum plants in the field [109]. Experiments with maize first showed no differences [110]. But in another experiment, freezing initiated some changes in the biochemical and hormone contents [111]. In tomato, only some insignificant differences between plants grown from frozen and unfrozen seeds were found [112]. Seedlings germinated from cryoconserved flax seeds initially developed better than those of the control variant, but afterwards they evened out [113]. Flow cytometry analyses of germinated orchid seeds after cryoconservation (both stored with and without a cryoprotector) detected no chromosome aberrations [114].

Analyses of available bibliographies show an evident lack of information about the consequences of seed storage in liquid nitrogen. This confirms the necessity of further investigations for the development of appropriate technologies for the long-term conservation of genetic resources. Previous experiments showed some differences between crops tested for their reaction to storage in liquid nitrogen. Therefore, it is important to evaluate a wider range of species.

Flax is not an ordinary plant. Several fundamental genetic findings were made on the basis of this species. *Linum usitatissimum* L. was one of the first plants with which Mendel’s laws were rediscovered in the beginning of the 20th century [115], etc. Also, while evaluating flax resistance to rust, H.H. Flor developed the theory “gene–for–gene” [116]. In addition to that, genotrophs were first described for flax [117]. Later, it was shown that this phenomenon is caused by DNA modification [118,119]. Therefore, according to its specific features, this species has been chosen for the analysis of the cryoconservation influence on plants’ agronomic characters.

Our previous experiments showed some negative, but not crucial, influence of cryoconservation and, following 6 months’ storage at room temperature (**RT**), on flax germination ability [120]. However, agronomical characters of adult plants have not been tested yet.

The current study investigated the influence of different seed storage temperatures on plant characteristics grown from these seeds and seeds of the next generation.

To reach this goal, agronomic characters of plants grown from seeds stored for different times under different conditions (different temperatures and different freezing rates) were compared.

## 2. Results

### 2.1. Germination Energy and Ability of Flax Seeds After Storage Under Different Conditions

**Figure 1:**
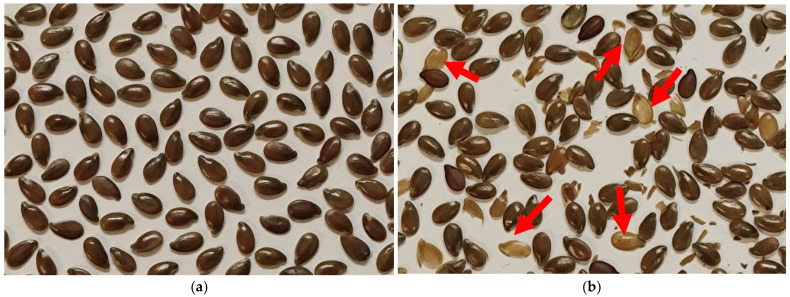
Flax seeds: (**a**) without any treatment; (**b**) damaged after gradual freezing in liquid nitrogen (red arrow).

The germination energy of seeds from the control variant (storage at **room temperature, RT**) was 98%. Following storage at all temperatures tested in this study—freezer (at **−10 °C** (**m10**), **−30 °C** (**m30**), **−50 °C** (**m50**), **−80 °C** (**m80**))—it remained practically the same (94–98%). At the same time, after storage in liquid nitrogen, germination reduced to 89% for **gradually frozen seeds** (**Ng**), and to 93% for seeds **directly immersed** (**Nd**) in liquid nitrogen in comparison with the control group. Also, some seeds had cracked seed coats after being removed from liquid nitrogen and thawed at **RT**. After gradual freezing (**Ng**), more severe seed coat damage was observed (Figure 1).

**Figure 2:**
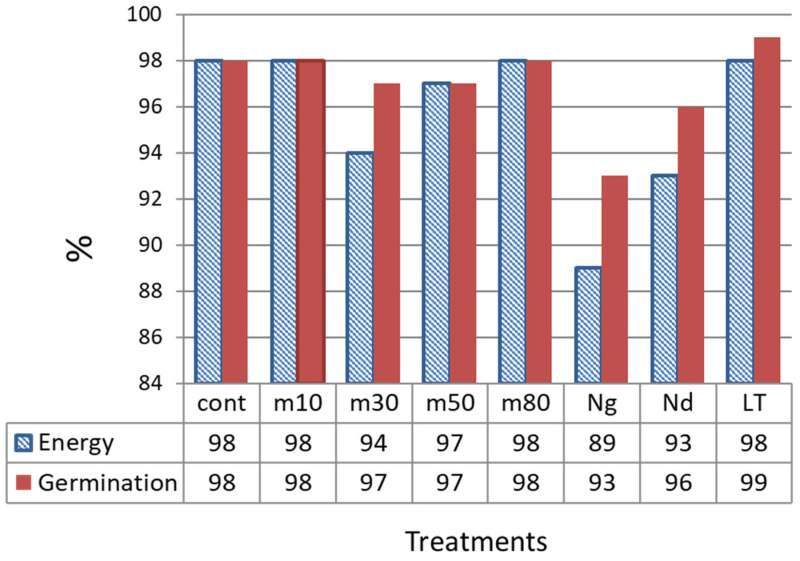
Germination energy and laboratory germination ability of the Orshanskiy-2 flax variety after different types of storage. Different types of seed storage: m10, m30, m50, m80—freezers at −10 °C, −30 °C, −50 °C, −80 °C, respectively. Nd—direct immersion in liquid nitrogen; Ng—gradual freezing in liquid nitrogen.

The laboratory germination ability was very close to that of the control variant: 96% for seeds after direct immersion in liquid nitrogen and 93% for seeds after gradual freezing. Seeds preserved at −10 °C for 24 years retained their viability, showing 98% germination energy and 99% germination ability (Figure 2).

### 2.2. Influence of Seed Storage Type on Characteristics of Plants Grown from Seeds Sown Immediately After Storage

#### 2.2.1. Overall Coefficient of Variation (OCV) of Flax Characteristics After Storage in Different Conditions

**Figure 3:**
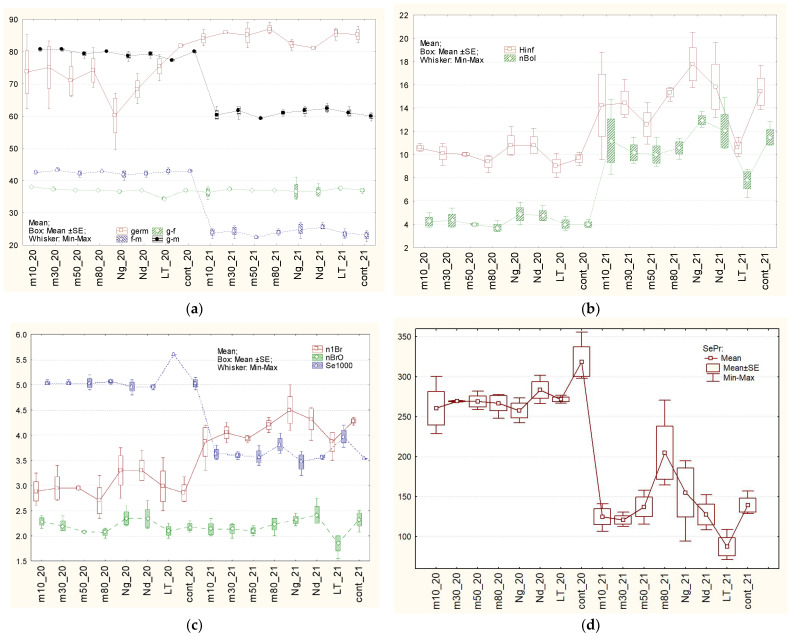
Average values of field germination ability, vegetative period phases, and seeds productivity parameters of plants grown after different types of storage in 2020 (_20) and in 2021 (_21). Different types of seed storage: m10, m30, m50, m80—freezers at −10 °C, −30 °C, −50 °C, −80 °C, respectively. Nd: direct immersion in liquid nitrogen; Ng: gradual freezing in liquid nitrogen. (**a**) Field germination ability (%); periods: germination—flowering (g–f), flowering—maturity (f–m), germination—maturity (days); (**b**) Length of the inflorescence (Hinf), cm; number of bolls on the plant (nBol); (**c**) Number of first-order branches (n1Br), number of branching orders in inflorescence (nBrO), mass of 1000 seeds (Se1000) g; (**d**) Seed yield (SePr), g.

**Figure 4:**
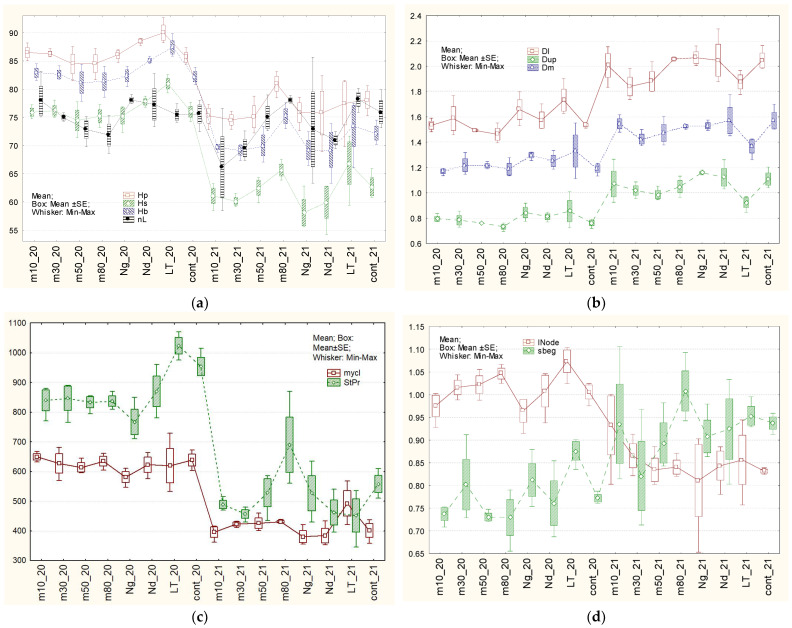
Average values of stem parameters of plants grown after different types of storage in 2020 (_20) and in 2021 (_21). Different types of seed storage: m10, m30, m50, m80—freezers at −10 °C, −30 °C, −50 °C, −80 °C, respectively. Nd—direct immersion in liquid nitrogen; Ng—gradual freezing in liquid nitrogen. (**a**) Total plant height (Hp), height to the first boll (Hb), height to the inflorescence (Hs), cm; number of leaves on the stem (nL); (**b**) Upper, medium, low stem diameter, height to the (Dup, Dm, Dl), mm; (**c**) Ratio height to the inflorescence to middle-stem diameter (mycl), Straw yield (StPr), g; (**d**) Length of internodes (INode), cm, difference between down- and upper-stem diameters (sbeg), mm.

**Figure 5:**
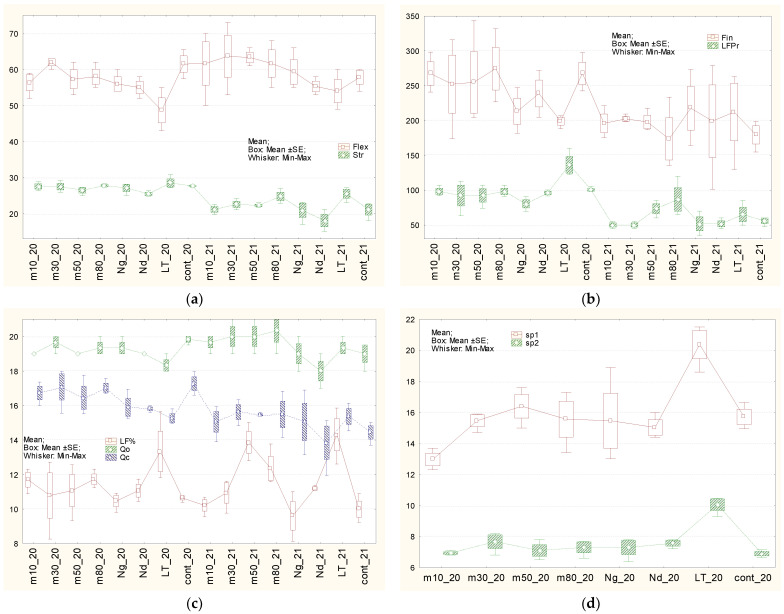
Average values of fiber properties of plants grown after different types of storage in 2020 (_20) and in 2021 (_21). Different types of seed storage: m10, m30, m50, m80—freezers at −10 °C, −30 °C, −50 °C, −80 °C, respectively. Nd—direct immersion in liquid nitrogen; Ng—gradual freezing in liquid nitrogen. (**a**) Fiber strength (Str), daN; fiber flexibility (Flex), mm; (**b**) Fiber fineness (fin), mm; long-fiber yield (LFPr), g; (**c**) amount of long fiber (%LF), organoleptically estimated (Qo) and calculated (Qc) fiber quality; (**d**) plant length from the top to snap point (sp1), length from the root to snap point (sp2) (cm).

For the classification of characters’ variability, different systems are used. According to the classification of H.V. Lorenzo et al. [120] (Table 1, Figure 3, Figure 4 and Figure 5), flax characters were divided into three groups. The first one, with low OCV—from 1.1 to 5.1%—included: duration of vegetative period stages (Figure 3a), total plant height, number of leaves on the stem (Figure 4a), length of the internodes (Figure 4d), middle diameter of the stem (Figure 4b), ratio between height to inflorescence and middle diameter of the stem (Figure 4c), mass of 1000 seeds (Figure 3c), calculated and tested organoleptically fiber quality (Figure 5c). A group with medium OCV—from 5.1 to 10.2%—included: field germination ability of seeds (Figure 3a), inflorescence length (Figure 3b), lower and upper diameters of stems (Figure 4b) and the difference between them (Figure 4d), number of inflorescence branches, degrees of inflorescence branching (Figure 3c), number of bolls (Figure 3b), straw yield (Figure 4c), long-fiber content (Figure 5b), seeds yield (Figure 4d), and fiber flexibility (Figure 5a). High OCV—from 10.2 to 16.4%—included: long-fiber yield, fiber fineness (Figure 5b), and stem length from the upper point to the snap point (Figure 5d). However, according to N.S. Rostova [121], the first two groups belong to low-variable ones (CV < 10%), and the third one to the intermediate group (25% ≥ CV > 10%).

So, the majority of tested characters did not demonstrate high variation after different regimes of storage.

#### 2.2.2. One-Way ANOVA Analyses of Flax Characters After Different Types of Seed Storage

Using ANOVA analyses allows identifying the reliable influence of seed storage conditions on adult plant characters and significant differences between types of seed storage.

**Figure 6:**
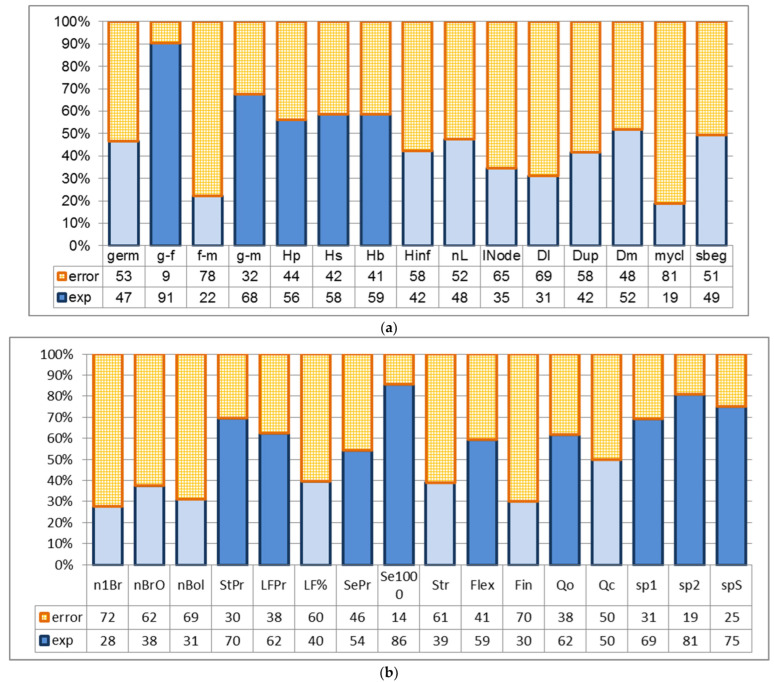
Influence of seed storage types on the manifestation of morphological, phenological, and economically valuable flax traits based on the results of one-way ANOVA (first year after seed treatment, average values of characters in 2020). (**a**) Field germination ability, duration of plant development stages, plant height parameters, inflorescence length, number of leaves, average internode length, stem diameters, ratio of stem length to middle-stem diameter (**mycl**) and difference between low and upper diameters (**sbeg**). (**b**) Characters of inflorescence structure, parameters of productivity, fiber quality and stem snap point. Influence of the storage treatments is significant when *p* < 0.05% (highlighted in dark blue). Plant traits are as described in Section 4.

One-way ANOVA showed a significant influence of seed storage types on the following evaluated flax characters: duration of the stages from germination to flowering (91%); from germination to ripening (68%); total height, height to inflorescence, height to the first boll (56–59%); straw yield (70%); fiber yield (62%); seed yield (54%); 1000-seed weight (86%); fiber flexibility (59%); organoleptically estimated fiber quality (62%); and also stem length to and the upper snap point, including stem length in the case of snap point estimation (69–81%) (Figure 6).

So, the type of seed storage significantly influences the manifestation of 14 flax characters out of 31 tested. These characters can be combined into four groups: (1) the period from germination till the beginning of flowering and, connected with it, the duration of the vegetative period; (2) plant height, straw and long-fiber yields connected with it, and also parameters of snap point; (3) fiber flexibility and, connected with it, parameters of fiber quality; (4) yield and weight of 1000 seeds.

For GeneBanks, differences between results of various storage types are not so important as their distinction from the control variant. Such dependency can be detected for characters that were influenced by the type of storage, using all three ANOVAs with post hoc comparison (HSD Tukey’s criteria) and the parametric-Student criterion (which is more powerful) or nonparametric Mann–Whitney U test.

#### 2.2.3. Influence of Seed Storage Types on the Manifestation of Flax Characteristics, According to the Results Obtained by Using the t-Student Criterion, Mann–Whitney U Criterion, and Tukey Criterion

Almost all tested types of seed storage did not have a significant influence on flax characters, except for the variant based on gradual freezing of seeds in liquid nitrogen, which was caused by mechanical damage to the seed coat, and also long-term storage, caused by the obsolescence and influence of low temperatures (Figure 3, Figure 4 and Figure 5, Table 1 and Table S1). Therefore, seed storage in liquid nitrogen with gradual freezing had a negative effect on straw (−186 g), long-fiber (−20 g), and seed (−61 g) production. In the case of long-term storage, in comparison with the control variant, the periods from germination to flowering and from germination to ripening became shorter by 3 days, plants grew taller by 4–5 sm, formed 70 g more straw and 36 g more fiber. At the same time, fiber flexibility reduced by 13 mm, and the organoleptically estimated fiber quality reduced by 1.5 points. Plants had longer stems from the top to the snap point and also from the snap point to the root. This may indicate that fiber formation began later in ontogenesis. This may have been related to flexibility, and, therefore, the organoleptically estimated quality of the fiber, or, conversely, stem growth, significantly outpaced fiber formation. The weight of 1000 seeds increased significantly, by 0.6 g. Storage in liquid nitrogen (gradual and direct immersion) may have affected field germination. Significant differences compared to the control were confirmed by the Student’s *t*-test (*p* < 0.01 and *p* < 0.02). According to the Mann–Whitney U criterion, values approached 0.05 (0.0495), and exceeded 0.05 (0.0537 and 0.43) according to the most stringent Tukey criterion. Other statistical differences did not make biological sense because they were caused by the high accuracy of measurements, which makes even small differences statistically significant.

Thus, seeds of the flax variety Orshanskiy-2 were weakly influenced by different storage regimes, which is favorable for GeneBanks. However, gradual freezing of seeds in liquid nitrogen raises concern in terms of possible damage to the seed coat. The same applies to long-term storage (24 years), which corresponds to the probability of genotrophs (long-term modifications) appearing [118,119].

### 2.3. Influence of Storage Type on the Characteristics of Flax Plants Grown from Seeds After One Reproduction (Long-Term Modification)

#### 2.3.1. Overall Coefficient of Variation of Flax Characters After Different Storage Treatments and Passage Through One Reproduction in the Field

According to OCV, characters were divided into three groups (according to [120]). Characters with “low” OCV ranged from 1.1 to 7.8% (germination ability in the field, duration of vegetative period phases (Figure 3a), plant heights, number of leaves on the stem (Figure 4a), length of internodes (Figure 4d), stem diameters (Figure 4b), difference between lower and upper stem diameter (Figure 4d), number of branches (Figure 3c), mass of 1000 seeds (Figure 3c), fiber flexibility (Figure 5a) and fineness (Figure 5b), organoleptically estimated and calculated fiber quality (Figure 5c)); “medium” OCV ranged from 7.9 to 15.6% (length of inflorescence (Figure 3b), ratio of stem height to inflorescence to its middle diameter (Figure 4c), number of inflorescence branching levels (Figure 4a), number of bolls (Figure 3b), straw yield (Figure 5a), content of long fiber (Figure 5c)); and “high” OCV ranged from 15.6 to 24.5% (yields of long fiber (Figure 5b) and seeds (Figure 3d)) (Table 2, Figure 3, Figure 4 and Figure 5). Thus, according to the presented grades of OCV, characters became more variable. According to general biological rules and classification suggested by N.S. Rostova [121], the first group and two characters from the second group—number of inflorescence branching orders and ratio of stem height to inflorescence to stem middle diameter—belong to the low-variable (CV < 10%) set, while the remaining eight characters from the second group and the entire third group belong to the intermediate set with 25% ≥ CV > 10%.

The majority of characters retained their status. Six characters (field germination ability, low- and upper-stem diameters, and their difference, number of main branches in the inflorescence, fiber flexibility) changed their medium OCV to low. Two characters—the ratio between stem height to inflorescence and stem middle diameter (**mycl**), and fiber strength (**Str**)—oppositely became low from medium. The OCV of seed productivity became high instead of medium. The position of fiber fineness changed from high to low. Therefore, the majority of characters display low variability between the storage results.

#### 2.3.2. One-Way ANOVA Analyses of Flax Characters After Different Types of Storage and One Cycle of Reproduction

**Figure 7:**
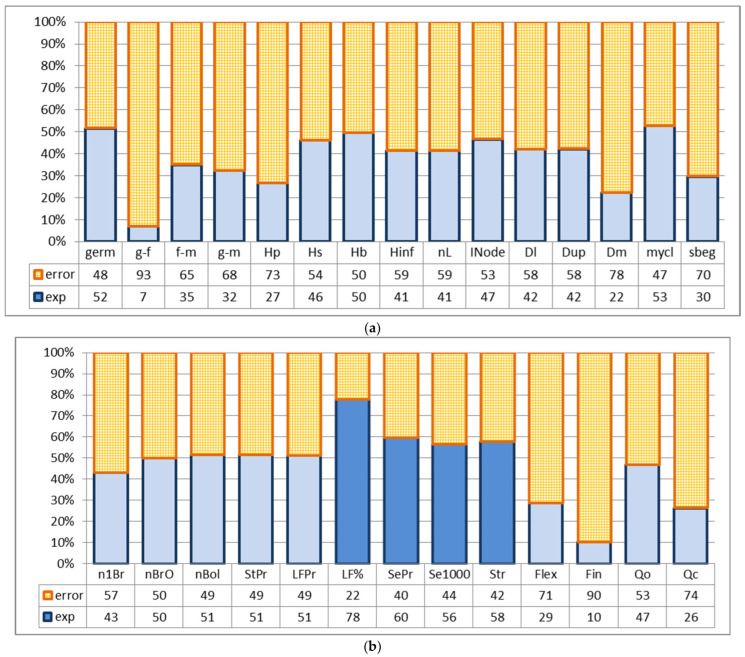
Influence of the seed storage types on the manifestation of morphological, phenological and economically valuable flax traits based on the results of one-way ANOVA (sowing with reproduced seeds, average values of characters in 2021). (**a**) Field germination ability, duration of plant development stages, plant height parameters, inflorescence length, number of leaves, average internode length, stem diameters, ratio of stem length to middle-stem diameter (**mycl**), and difference between lower and upper diameters (**sbeg**). (**b**) Characters of inflorescence structure, parameters of productivity and fiber quality. Influence of the storage treatments is significant when *p* < 0.05% (highlighted in dark blue). Plant traits are as described in Section 4.

One-way ANOVA analysis revealed a high influence of different types of seed storage on seed production (portion of influence—60%), mass of 1000 seeds (56%), fiber output (78%) and its strength (58%) (Figure 7).

Analyses showed a significant influence of storage type on 4 out of 28 evaluated characters. Among these, seed characteristics confirmed their dependence on the type of storage after seed reproduction. Fiber strength in the first year of the experiment showed a tendency to change its manifestation after long-term storage and preservation at −80 °C, which became significant the following year. A similar tendency to change in the first year of evaluation was demonstrated by fiber content after long-term storage. The following year, this difference became significant. Differences after storage at −50 °C were expressed only in the next generation. Therefore, these results need further confirmation.

For GeneBanks, even a tendency of prolonged influence of storage type on plants’ characters serves as a signal for modification or even rejection of such storage methods. That is why special attention is paid to the difference between results of a definite type of storage and the control.

#### 2.3.3. Influence of Seed Storage Types on the Manifestation of Flax Characters After One Seed Reproduction, According to the Results Obtained by Using the t-Student Criterion, Mann–Whitney U Criterion, and Tukey Criterion

Different types of storage demonstrated various influence on different characters (Figure 3, Figure 4 and Figure 5, Table 2 and Table S2). After **long-term storage** and 2 years of subsequent reproduction, the mass of 1000 seeds increased by 0.5 g, and in 2020, it increased by 0.6 g (significantly). The long-fiber content increased by 4.3%, though in 2020 it increased by 2.7% (insignificantly). Fiber yield increased significantly, and its content also increased but was on the border of significance. Fiber-breaking load in 2021 increased to the border of significance by 4.3 daN, though in 2020 this increase was on the border of significance. Seeds yield in 2021 reduced by 53 sg in comparison with 2020, when it reduced by 47 g, on the border of significance. The influence of seed storage in **liquid nitrogen after gradual** freezing on flax characters was not detected. A possible reason for this could have been damage to the seed coat in the first year after storage. Storage at −50℃in 2021 increased fiber content by 3.9%, though in 2020, it increased by 0.5%—insignificantly. Other differences detected in 2020 did not repeat. The remaining differences detected in 2021 mainly have statistical, but not biological, reasons because they are based on the high accuracy of measurements, which makes even small differences significant.

The seeds of flax variety Orshanskiy-2 demonstrated low susceptibility to different storage types and temperatures, which is favorable for their preservation in GeneBanks. On the other hand, some differences associated with long-term storage (24 years at low temperature) were confirmed. This is consistent with the phenomenon of genotrophs—long-term modifications described for flax [117,118,119]. Also, **gradual** freezing **in liquid nitrogen** is not recommended because it leads to seed damage.

### 2.4. The Influence of Seed Storage Type and Year of Reproduction After Storage on Flax Plant Characteristics

#### The Influence of Seed Storage Type, Reproductive Cycle After Storage, and Their Interaction on Flax Plant Characteristics, According to ANOVA Analysis

The most important data was obtained when the results of two cycles of reproduction were compared. As conditions in 2020 and 2021 differed, it is impossible to compare characteristics directly. That is why we used the method of “reduced average values” [122], which we developed for comparison of plant accessions grown in different years. The method is based on the comparison between the percentages of characteristic values in relation to a uniform standard, which is cultivated every year, and multiplied by the long-term average value of this standard’s characteristic. In the current case, 97% of the standard’s value for the period from germination to flowering duration is one day (experiment—36 days, control—37 days), which means an insignificant difference. At the same time, a difference of three days (92% of the control) is significant.

**Figure 8:**
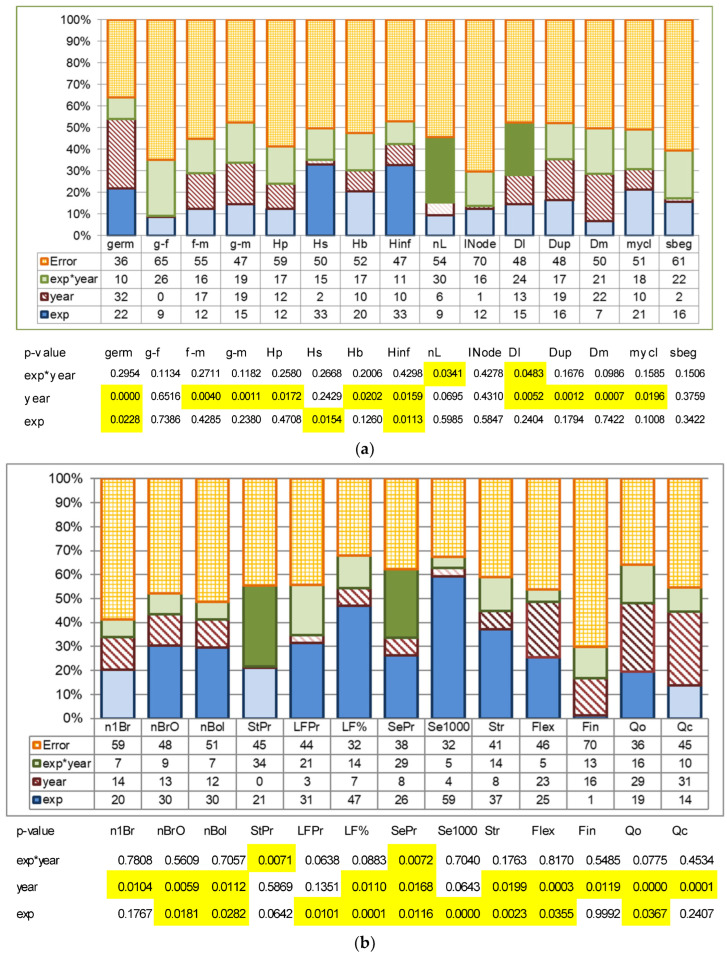
Influence of storage type, seed reproduction cycles and their interaction on the manifestation of morphological, phenological, and economically valuable flax traits based on the results of a two-way ANOVA (reduced average values of characters in 2020 and 2021). Where: exp—is a type of experiment; year—is the year of reproduction or influence on the next reproduction; exp * year—the interaction of experiment type and generation of reproduction. (**a**) Field germination ability, duration of phenological phases, plant height parameters, inflorescence length, leaf number, internode length, stem diameters, ratio of stem length to middle-stem diameter (**mycl**), and difference between lower and upper diameters (**sbeg**). (**b**) Characters of inflorescence structure, productivity and fiber quality. The influence of the storage treatments is significant when *p* < 0.05% (highlighted in dark blue). The influence of the reproduction year is significant when *p* < 0.05%, marked by dark shading. Influence of the interaction between storage treatments and year of reproduction is significant when *p* < 0.05% (highlighted in dark green). Plant traits are the same as described in Section 4.

Using reduced average values, we completed a two-way ANOVA analysis, which included the following factors: (1) type of experiment; (2) year of reproduction—in our case it was influence of the next reproduction; and (3) the interaction of experiment type and generation of reproduction (Figure 8).

The type of the experiment displayed its influence on germination ability (22%), height to inflorescence (33%), length of inflorescence (33%), number of branching orders in inflorescence (30%), number of bolls (30%), fiber yield (31%), fiber content (47%), seed yield (26%), mass of 1000 seeds (59%), fiber strength (37%), fiber flexibility (25%), and organoleptically estimated fiber quality (19%).

The year of the experiment (reproduction) significantly influenced field germination ability (contributing 32%); the duration of periods from flowering to maturity (17%) and from germination to maturity (19%); total plant height (12%); and height to the first boll (10%); inflorescence length (10%); down-, middle- and upper-stem diameters (13–22%); the ratio between stem height to inflorescence and middle-stem diameter (10%); the number of first-order branches (14%); number of branching orders in the inflorescence (13%); the number of bolls (12%); fiber content (7%); seed yield (8%); fiber flexibility (23%); fiber strength (8%); fiber quality estimated organoleptically (29%); and calculated fiber quality (31%).

The interaction of the experiment type and generation of seeds’ reproduction (first–second) was revealed for number of leaves on the stem (*p* = 0.034, portion of influence—30%), low-stem diameter (0.048, portion of influence 24%), straw yield (0.007, portion of influence 34%), and seeds yield (0.007, portion of influence 29%).

The interaction of the experiment type and reproduction order was detected for number of leaves (*p* = 0.034, portion of influence—30%), low-stem diameter (0.048, portion of influence 24%), straw yield (0.007, portion of influence 34%) and seed yield (0.007, portion of influence 29%).

Post hoc analyses according to HSD Tukey did not reveal any change in expression for the number of leaves on the stem, but a weaker Fisher’s LSD criterion showed a significant reduction in their number (after storage at −10 °C) in the 2021 reproduction (66 leaves) and the majority of other treatments of the experiment (75–78 leaves), and also after storage at −30 °C in 2021 (69 leaves) in comparison with some other types of storage (78 leaves) (Table S3).

Post hoc analyses according to HSD Tukey showed significant differences (increase) in low-stem diameters between plants grown from seeds sown straight after long-term storage in 2020 (2.02 mm) and plants grown after reproduction in 2021 after storage at −30 °C, −50 °C, and long-term storage (1.61–1.64 mm). These analyses also showed a significant increase in straw yield in plants grown after storage at −80 °C and reproduction in 2021 (934 g) in comparison with some other variants (607–625 g), and also in seed yield after the same type of storage and the majority of other storage types (143–210 g) (see Table S3).

A significant influence of the interaction between the type of experiment and the year of seed reproduction on character manifestation was not detected. At the same time, some storage variants reduced seed germination ability.

A statistically significant influence of interaction between types of seed storage and year of reproduction on evaluated characters was not detected. At the same time, several characters changed their expression after some storage treatments. In 2020, the field germination ability of seeds after gradual freezing in liquid nitrogen was 60%. Seeds from other variants had a germination ability of 80–85%. Plants grown from seeds after long-term storage and one reproduction had a higher content of long fiber (14.8%) in comparison with other experiments (9.7–11.3%). The yield of long fiber and straw after storage at −80 °C and reproduction in 2021 increased up to 121 g/m^2^ in comparison with 62–70 g/m^2^ in other variants. Interesting results were obtained for a very conservative character—weight of 1000 seeds. Seeds of both first and second generations after long-term storage increased it up to 4.8 g, though in other variants it was 4.3 g (see Table S3).

Thus, two-way ANOVA analysis confirmed the basic conclusions suggested after one-factor analyses carried out previously, but also revealed new patterns concerning the increase in straw, long-fiber, and seed yields after some storage treatments (−50 °C, −80 °C, long-term storage), which need further investigation.

## 3. Discussion

### 3.1. Germination Energy and Ability of Flax Seeds After Storage in Different Conditions

Two-factor variance analyses (type of freezing, year of reproduction) displayed the influence of both factors on germination ability in the field. At the same time, differences between field and laboratory germination ability tested in the lab were not detected between seeds stored at different temperatures, −10, −30, −50, −80 °C, and also during long-term storage at −10 °C. That is, freezing temperature does not affect seed viability. At the same time, there is a tendency for field germination ability to be reduced by 6–11% compared to the control variant. Flax seeds can preserve their viability at −10 °C for at least 24 years. However, storage in liquid nitrogen can lead to the reduction of laboratory germination ability and energy, and especially field germination ability (by 14–22%). The biggest loss of viability was associated with gradual freezing in liquid nitrogen, which resulted in seed coat damage.

The germination ability of seeds produced by plants grown from frozen seeds showed no difference compared to the control group, indicating that liquid nitrogen has only a mechanical freezing effect and does not influence the viability of next-generation seeds.

### 3.2. Modification of Flax Characteristics Immediately After Freezing and in Subsequent Seed Reproduction

As flax is a crop for which genotrophs have been described [117,118,119], a wide range of plants’ agronomic characteristics were analyzed in two generations.

In the first year, contrary to expectations, the range of plants’ agronomic characteristics’ variability between different types of storage was lower than that of reproduced plants. The highest OCV was 16.4%, which, according to N.S. Rostova [121], refers to low average variability of characteristics. Characters with high OCV include only long-fiber yield, fiber fineness, and stem length from the upper point to the snap point, that is, characteristics with their own high variability.

One-way ANOVA and the strictest post hoc Tukey criterion showed a significant influence of storage type on 14 out of 31 evaluated characters, including the period from germination to the beginning of flowering and, connected with it, the duration of the vegetative period; plant height; straw and long-fiber yields; and also parameters of the snap point, fiber flexibility, and, connected with it, parameters of fiber quality, yield, and the weight of 1000 seeds. To avoid discussion of the border-line values, we used the strictest criteria to detect storage treatment conditions that significantly influence characters’ expression.

The most evident changes in flax characteristics were detected after **gradual freezing** in liquid nitrogen, which was caused by mechanical damage to the seed coat, and **long-term storage**, caused by the deterioration and influence of low temperatures. Therefore, seed storage in liquid nitrogen with **gradual freezing** had a negative effect on production characteristics (straw, long-fiber, and seed yield), which is expected because of the reduction in plant number. In the case of **long-term storage,** compared to the control variant, differences are higher and cannot be explained by low germination ability. After this type of storage, compared to the control, the duration of the germination–flowering period and the associated whole vegetative period were reduced. Plants grew taller, which increased straw and fiber yield but reduced the organoleptically detected fiber quality and its flexibility. Plants had longer stems from the top to the snap point and also from the snap point to the root, which probably influenced fiber quality.

Plants of the next generation increased the range of variability. In 2020, the maximum OCV was 16.4%, and in 2021, this level appeared to be the lowest (15.6–24.5%). Straw yield confirmed its high variability, but fiber fineness became less variable. Seed yield became highly variable, possibly because of highly unfavorable weather, and the yield reduced by half (total average yield in 2020—275 g, in 2021—137 g).

In 2021, a significant influence on seed production and the 1000-seed weight was preserved. A significant influence of storage type on fiber content and breaking load was also noted. Different storage types demonstrated varying influences on different characteristics.

After **long-term storage,** in comparison with the control variant in 2021, an increase of 1000 seeds’ weight was preserved. The increase in fiber yield became insignificant. Conversely, a long-fiber content increase (which was visible in 2020, but insignificant) was confirmed. The fiber-breaking load increased to a significant level. However, seed yield was reduced.

The influence of storage in **liquid nitrogen after gradual freezing** on agronomical characters was not detected. A possible reason for this could have been damage to the seed coat.

Storage at −50 °C significantly increased fiber content in comparison with that detected in 2020. Other differences detected in 2020 did not repeat.

Two-factor variance analyses using the reduced average values [122] showed that the type of experiment influences the expression of 12 out of 28 tested characters: germination ability, height to inflorescence, length of inflorescence, number of branching orders in inflorescence, number of bolls, fiber yield and content, seed yield, mass of 1000 seeds, fiber strength, flexibility, and organoleptically estimated and calculated fiber quality.

The year of the experiment (reproduction) significantly influenced 20 out of 28 characteristics: field germination ability; duration of flowering—maturity and germination—maturity periods; total plant height; and height to the first boll; length of inflorescence; down-, middle- and upper-stem diameters; ratio of height to the inflorescence to middle-stem diameter; number of first-order branches; number of branching orders in the inflorescence; number of bolls; fiber content; seed yield; fiber flexibility; fiber strength; and organoleptically estimated and calculated fiber quality.

Both factors influenced 9 out of 28 characters independently: germination ability, length of inflorescence, number of branching orders in inflorescence, number of bolls, fiber content, seed yield, fiber strength and flexibility, and organoleptically estimated fiber quality. These are characters that preserve the same tendency (even small and insignificant) of changing in both years of evaluation.

Interaction of the experiment type and seed generation influenced the number of leaves on the stem, stem diameter, straw yield, and seed yield, with a contribution of approximately 30%. These are traits that shifted the modification direction: they increased in 2020 and decreased in 2021, or vice versa.

### 3.3. Peculiarities of Long-Term Storage

Flax variety Orshanskiy-2, after long-term storage, mobilized its potential and became earlier, higher, producing a higher yield of straw, increased fiber content and yield. However, regarding the position of the snap point, fiber was finally formed later, then in plants grown from fresh seeds. This was a possible reason for the decrease in fiber flexibility and organoleptically estimated quality, and perhaps also for the reduction of the fiber fineness and calculated quality parameter (insignificant). Seeds of the first reproduction increased the mass of 1000 seeds, but the yield reduced (though insignificant). The following year, seeds of the second reproduction preserved a high mass of 1000 seeds, but their yield was reduced in comparison with the control variant. Fiber content increased and slightly increased its yield, which follows the tendency of the previous year. Fiber-breaking load increased to a statistically significant level. Unfortunately, in 2021 we did not have the opportunity to analyze the position of the snap point.

These results show the tendency of the attenuation of the storage time influence on flax characteristics. However, it is unknown how many years this process will take, and it is better to preserve seeds in different conditions.

### 3.4. Which of the Seed Storage Treatments Are to Be Used in GeneBanks?

Plants grown from flax seeds after 24 years’ storage at t° −10 °C have been found to express significant changes in the duration of vegetative period phases, productivity and some quality characteristics. Some, but not all of these changes, attenuate already the next year. But generally, it will take more time. So, it is not recommended to preserve flax seeds only in such storage. It is better to have storage facilities with different conditions.

It is necessary to carry out additional detailed evaluation of the consequences of seed storage at −50 °C.

Due to a lack of data, we cannot recommend long-term storage at other temperatures.

### 3.5. Perspectives

Long-term character modifications after long-term storage may be caused by changes in the methylation level of genes, which control these characters [47,53]. It will be useful to evaluate the level of DNA methylation in seeds immediately after long-term storage, and after the first and second reproduction, and compare these results with DNA methylation of DNA in adult plants using MSAP (methylation-sensitive amplification polymorphism) [47]. To reduce the negative influence of liquid nitrogen on seeds, it is recommended to use rapid cooling and/or cryoprotectors, which have been successfully used for other crops, after preliminary experiments [88,91,95,102,104,105,106].

## 4. Materials and Methods

### 4.1. Plant Material

For the experiments, seeds of the fiber flax variety Orshanskiy-2 were used. It was bred in 1969 at the Byelorussian Institute of Agriculture and included in the USSR National catalog in 1971. This variety has fiber of good quality and high spinning ability, medium fiber content—18.5–22.6%, and is resistant to lodging. To this day, it is used in our experiments as a standard of fiber quality. Seeds of the Orshanskiy-2 flax variety, multiplied and harvested in August 2019, were used. Initially, they had a germination rate of 98% and a moisture content of 6.3%, tested 3 days before packaging for storage according to Russian State Standards. Moisture content was determined using a gravimetric-based method on a moisture analyzer MA 100 Sartorius using the “high-constant-temperature” drying method at a temperature of 130 °C for 1 h (results are comparable with the ISTA reference method) [123,124,125].

### 4.2. Variants of Seed Storage Treatments

Flax seeds reproduced in 2019 were stored under different conditions. Seed samples of 10 g (=1923 seeds) were used for each variant. From the beginning of March 2020 to the end of April 2020, seeds were stored in different conditions. That is, the seeds were stored under controlled conditions for 60 days. As a control (**cont**), seeds preserved at room temperature (**RT)** in a paper bag after harvesting in August 2019 and during the whole period of the experiment were used.

Different storage treatments were employed. Seeds were placed directly in freezers at −10 °C (**m10**), −30 °C (**m30**), −50 °C (**m50**), −80 °C (**m80**), and in liquid nitrogen (−196 °C-cryopreservation). The last type of storage was carried out in two treatments:

1. **Direct immersion (Nd)** in liquid nitrogen. Bags with seeds were immediately transferred to bioproduct storage.
2. **Gradual freezing (Ng)**. Bags with seeds were slowly cooled (0.80 °C per 1 min) during 2 h until reaching −70 °C (in program freezer) and then immediately placed in liquid nitrogen.

Seeds in bags were stored in liquid nitrogen in biological product storage vessels HB-0.5 with volume of 0.5 m^3^ [126].

For freezing and cryopreservation, seed samples were put in paper bags and then in aluminum foil packages. The edges of the bags were bent several times and fixed with a stapler, which ensured hermetic packaging.

Additionally, seeds stored for 24 years (reproduced in 1996) in hermetic aluminum foil bags at −10 °C were taken from the special storage (**LT**).

Because all variants of the experiment were stored in sealed bags, the humidity of the air was not determined during storage.

In May 2020, three days before sowing, seeds from all treatments were taken from storage and thawed. Seed laboratory germination ability and germination energy were determined on a paper substrate made of filter paper at +20 °C in a thermostat, in the dark, taking into account the percentage of germinated seeds on day 3 (germination energy) and day 7 (germination). The seeds were considered germinated when the germinal root appeared longer than the seed’s length. The main experiment was focused on a field study (3 replications of 7 experimental variants for about 2000 seeds), so laboratory determination was done to control 100 seeds without repetition. After evaluating the seed quality in the laboratory, the remaining seeds were sown in the field.

Seeds, harvested from the plots, cultivated in 2020, were stored until the next season at **RT** in paper bags. In May 2021, they were sown in the field for further evaluation.

### 4.3. Field Experiments

#### 4.3.1. Place of the Field Experiments

Field experiments were carried out on the field of VIR in the Leningrad region, which is situated near the Baltic Sea at latitude 60° North. In June, daylight reaches 20 h. During the vegetative period, the average temperature is 14 °C, and total precipitation is 350 mm. Soils of this region are brown podzolic, light adobe, with a humus content of 3–4% and a pH of 5.5–6.0.

#### 4.3.2. Weather Conditions During the Field Experiments

**Figure 9:**
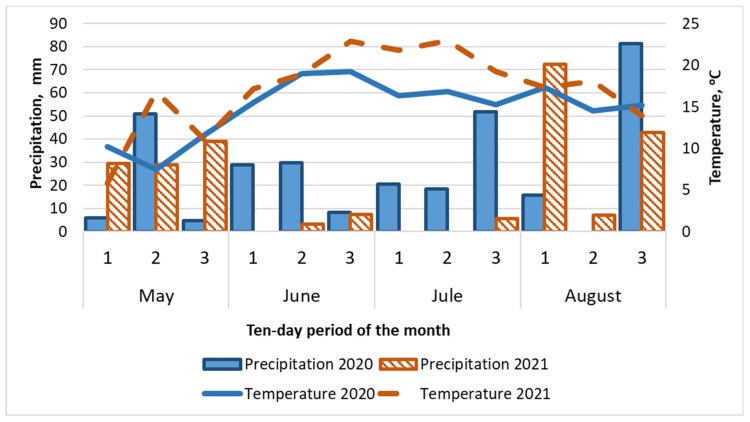
Ten-day period of the month average air temperature and the amount of precipitation in Leningrad region (2020, 2021).

In the middle of May 2020 and 2021, seeds from all storage treatments were sown simultaneously. Meteorological conditions were documented by a meteorological station situated straight on the experimental field. Weather conditions of flax vegetative periods in both years differed considerably, which can be seen in Figure 9. In 2021, it was rather warmer, but significantly dryer than in 2020. In both years, the soil had enough water for rapid and uniform seed germination. In 2020, the period of seed germination, which usually happens in the beginning to middle of May, fell during cold and rainy weather. And in 2021, this period was much warmer. In 2020, flowering happened in rather warm and rainy weather. In 2021, the period of flowering and early ripening appeared to be relatively dry. Only the beginning of August was rainy. As a whole, both years of the experiment were favorable for flax cultivation; since the temperature during the growing season was within the normal range, there was no spring drought, and there was no excessive precipitation.

#### 4.3.3. Field Evaluation

Seeds from each variant of storage were sown in three replicates (1923 seeds per replicate) on plots of 1 m^2^, with spacing between rows of 8 cm. Plants were grown and evaluated according to standard methods [127,128].

##### Testing of Seeds Germination Ability and Energy

Field germination ability, % (**germ**), was estimated by calculation of the germinated plants at the herringbone stage [127,128].

##### Evaluation of Vegetative Period Phases

To estimate the influence of different types of storage on the dynamics of plant development, the durations of vegetative period phases were tested (Table 3):

- Period from full germination, when 75% of seeds have germinated, to full flowering, when 75% of plants are flowering), days (**g-f**);
- Period from full flowering until the early yellow-ripening stage, when half of the bolls on the plot are yellow and dry, days (**f-m**);
- Period from germination to ripening, days (**g-m**);
- Period from sawing till ripening, days (**s-m**).

##### Evaluation of Morphological Characters

All plots were harvested at the yellow-ripening stage. Before harvesting, 20 typical plants were selected for further individual measurements. This is important to analyze characters playing the main role in yield formation and seed and fiber quality. The description of the inflorescence structure, which gives an idea about its influence on seeds yield, included evaluation of plant structure (Table 3):

- Total plant height, sm (**Hp**);
- Height to inflorescence, sm (**Hs**);
- Height to first boll (**Hb**);
- Inflorescence length, cm (**Hinf**);
- Number of leaves on the stem (**nL**);
- Average length of internodes, cm (**INode**);
- Number of the main branches in the inflorescence (**n1Br**);
- Number of inflorescence branching orders (**nBrO**);
- Number of bolls (**nBol**).

The description of the stem architecture is important, because it was discovered that fiber of the best quality is formed in long thin stems with the most even diameters [129]. For the stems’ architecture characterization, the following characteristics were measured:

- Ratio Hs/Dm (**mycl**);
- Difference between low- and upper-stem diameter (**Dl-Dup**), mm (**sbeg**).

##### Evaluation of Seed and Straw Productivity

After harvesting, all sheaves were placed to the mesh shed for 3 weeks until full ripening. After this, samples were threshed individually. Further were measured:

- Seed production, g/m^2^ (**SePr**);
- Weight of 1000 seeds, g (**Se1000**);
- Straw production g/m^2^ (**StPr**).

##### Evaluation of Fiber Productivity and Quality

Fiber was extracted after dew retting [130,131]. Dew retting is a method of primary processing of flax, when its stems are spread in a thin layer in the field. Flax straw is exposed to microorganisms, as a result of which the technical flax fiber is easily separated from the wood during its further processing on milling equipment.

Fiber content and its quality were detected according to standard guidelines [130,131]. Retted stems were hackled to separate long fibers **(LFPr**), and their percentage (**%LF**) was calculated relative to the straw weight. The technical flax fibers were combed on steel combs by hand with a different number of needles per unit area [132,133].

After this, parameters of long-fiber quality were tested. Fiber flexibility (**Flex**) measurements were made after special fiber preparations of 15 individual strands. A sample 27 cm long and weighting 420 mg was cut from the middle of each fiber strand. All preparations were aligned under the press for 8–10 h at 20 °C and 60–65% humidity. Fiber flexibility (mm) was measured according to the free flexure of both strand ends.

Fiber-breaking load (Newtons), that is strength (**Str**), was measured by means of dynamometer using the same 15 samples (27 cm length and 420 mg weight) for each sample. It is important to mention that the breaking-load data of the technical fibers indicates a value of comparative difference between the samples, allowing an estimation of the technical fibers’ strength, but did not give any information about the strength of elementary fibers.

For the measurement of fiber fineness (**Fin**), each fiber strand was carded on a special hatchel (10 needles/cm). A 10 mm length was cut from the middle of the strand. Three preparations of 10 mg were taken to count the number of fibers (n) in each preparation. Then, the average number of fibers per preparation was calculated. Average length of 1 mg of fiber was considered as (10 mm × n/10). Finally, fiber fineness was estimated in m/g.

Two general fiber quality parameters of technical fibers were assigned to the tested samples. One of them (**Qo**) was estimated organoleptically by experts, taking into account the length, color, brightness, fineness, flexibility, and strength of fiber. Another one (**Qc**) was calculated from the three described parameters using Formula (1).

**Qc** = 0.2 × **Str** + 0.1 × **Flex** + 0.013 × **Fin** + 2.1 (1)

##### Evaluation of Stem Snap Point Parameters

During plants’ vegetative growth (during the stage of fast growth), another interesting characteristic—the position of the snap point on the flax stem—was measured according to [134]. Snap point is a specific region in the growing flax stem, below which the bast fiber-enriched peels suddenly become much stronger than above it. It is a region where cells stop their elongation and cell wall thickening starts. According to the position of the snap point on the stem, the following characteristics were measured using 5 plants:

Length from cotyledons to snap point, cm (**sp1**);

Length from stem apex to snap point, cm (**sp2**);

Length from cotyledons to apex, cm (**spS**).

According to [134], the position of the snap point regarding the apex of the stem (**sp2**) remains constant during the fast growth phase of plant development, when the length from cotyledons to snap point (**sp2**) and length from cotyledons to apex (**spS**) remain constant. That is why it is important to analyze all three characteristics as circumstantial indicators of the fiber elongation end.

All characteristics (being in bold in the text) used in the current investigation or for statistical analyses are listed in Table 3.

### 4.4. Statistical Analysis

Statistical analyses of the obtained results were carried out using standard methods [135,136,137,138]. Basic statistics (mean, standard error, OCV) were calculated using MS Excel 10.0. The influence of the genotype and year conditions on the expression of flax characters was evaluated using two-factor ANOVA in the Statistica 7.0 program [139]. The effect size of the factor’s influence on characters was estimated using Fisher’s Formula (2):

ŋ^2^ = SSfactor/SStotal × 100% (2)

Differences between the results of storage treatments were estimated using one-way ANOVA, Tukey test, at *p* < 0.05.

Significance of differences among variants was analyzed using three criteria: Student’s *t*-test (the most usually used parametric criterion); Mann–Whitney U test (nonparametric test, the gentlest one); and Tukey’s HSD (honestly significant difference) test (parametric test, which uses the results of one-way ANOVA Post hoc comparison—the strictest criterion). The significance level for all criteria was *p* < 0.05 [139].

For the estimation of characters‘ variation level depending on the type of the experiment, the overall coefficient of variation (OCV) was used according to [120]; see Formula (3).

OCV = Standard deviation/Average × 100%(3)

To calculate this coefficient, average values from eight treatments were compared. The greater the difference between the compared averages, the greater the overall coefficient of variation. OCV had 3 levels: “high”, “medium”, and “low”, depending on the difference between the highest and lowest OCV of all characters divided by three. Each third part corresponded to its own level (according to [120], see Formula (4)).

A = (Max OCV recorded—Min OCV recorded)/3 Classification of the OCVs: “low” from Min OCV recorded to A %; “medium” from A to 2A; “high” from 2A to Max OCV recorded(4)

For the comparison of flax plant characters manifested in both years (2020 and 2021), the method of “reduced average values”, developed by the authors, was used [122]. It is based on the comparison of characters displayed in different years and expressed as a percentage of the union standard, which is grown each year. This percent can be recalculated into an average value of the tested genotype’s character. This method makes the results more visual.

Reduced average value was calculated by Formula (5):

X_reduced average_ = X_av.st._*X_av.%_/100, where X_reduced average_—reduced average value of the tested accession character; X_av.st._—multiannual average value of the standard accession character; X_av.%_—average % of character expression in the tested accession. (5)

## 5. Conclusions

Comparison of different flax seed storage conditions showed some variances in their influence on germinating ability and agronomic character expression in plants grown from preserved seeds and seeds of the next generation. First of all, it was discovered that flax seeds can preserve their viability at −10 °C for at least 24 years. Storage at −10 °C, −30 °C, −50 °C, and−80 °C did not influence seed germinating ability in laboratory tests, but a tendency of field germinating ability reduction was revealed. Gradual freezing in liquid nitrogen caused the destruction of the seed coat and reduction of germinating ability, especially when sown in the field. However, seeds harvested from the surviving plants had the same germinating ability as in the control variant. Plants grown after different storage variants significantly changed (increased or reduced) 14 out of 31 evaluated characters. The maximal differences were, as expected, distinguished after seeds’ gradual freezing in liquid nitrogen. Also, character modifications were found after long-term 24-year storage, caused by obsolescence and the influence of low temperatures. Possibly, long quiescence mobilized plant potential, and they became earlier maturing, taller, produced a greater yield of straw and fiber, and increased the mass of 1000 seeds. But fiber was finally formed later than in plants grown from fresh seeds, which could be the reason for its quality reduction. Plants of the next generation, grown from seeds harvested in 2020, reduced seed yield but preserved their size. The content of fiber and its yield increased slightly, which follows the tendency of the previous year. These results demonstrate the tendency of attenuation of storage time and conditions’ influence on flax characters. At present, it is unknown how many years (generations of seed reproduction) are needed for the complete elimination of such consequences of low-temperature seed storage. So, it is better to preserve seeds in different conditions.

## Figures and Tables

**Table 1:** Variation in characteristics of flax plants grown in field trials after different types of seed storage.

Trait ^1^	Cont ^2^	m10	m30	m50	m80	Ng	Nd	LT	OCV, % ^3^	OCV, Level ^4^
germ	81.9 ± 0.1 a	73.6 ± 6.6 a	75.1 ± 6.5 a	70.9 ± 4.5 a	74.2 ± 3.6 a	6.2 ± 5.3 a	68.3 ± 2.7 a	75.5 ± 2.4 a	8.7	Medium
g-f	37.0 ± 0.3 bc	38.0 ± 0.0 c	37.3 ± 0.3 bc	37.0 ± 0.0 bc	37.0 ± 0.0 bc	36.7 ± 0.3 b	37 ± 0 bc	**34.3 ± 0.3 a ^5^**	2.9	Low
f-m	43.0 ± 0.3 a	42.7 ± 0.3 a	43.3 ± 0.3 a	42.3 ± 0.7 a	43.0 ± 0.0 a	42.0 ± 1.0 a	42.3 ± 0.7 a	43.0 ± 0.6 a	1.1	Low
g-m	80.0 ± 0.3 b	80.7 ± 0.3 b	80.7 ± 0.3 b	79.3 ± 0.7 ab	80.0 ± 0.0 b	78.7 ± 0.9 ab	79.3 ± 0.7 ab	**77.3 ± 0.3 a**	1.4	Low
Hp	85.7 ± 0.9 ab	86.5 ± 0.9 ab	86.3 ± 0.4 ab	84.5 ± 1.7 a	84.5 ± 1.6 a	86.1 ± 0.7 ab	88.6 ± 0.4 ab	90.0 ± 1.4 b	2.2	Low
Hs	76.0 ± 1.0 ab	75.9 ± 0.7 ab	76.2 ± 1 ab	74.5 ± 1.8 a	75.2 ± 1.2 a	75.3 ± 1.5 a	77.8 ± 0.6 ab	81.0 ± 0.9 b	2.7	Low
Hb	82.2 ± 0.9 ab	82.9 ± 0.9 ab	82.6 ± 0.8 ab	81.2 ± 1.9 a	81.3 ± 1.6 a	82.4 ± 1.1 ab	85.1 ± 0.4 ab	87.4 ± 1.2 b	2.5	Low
Hinf	9.7 ± 0.3 a	10.6 ± 0.2 a	10.1 ± 0.5 a	10.0 ± 0.1 a	9.3 ± 0.5 a	10.8 ± 0.8 a	10.8 ± 0.7 a	9.0 ± 0.6 a	6.7	Medium
nL	75.6 ± 1.6 a	78.0 ± 2.6 a	75.0 ± 0.4 a	72.9 ± 1.6 a	71.9 ± 2.0 a	78.1 ± 0.5 a	77.3 ± 2.8 a	75.5 ± 1.0 a	3.0	Low
INode	1.01 ± 0.02 a	0.98 ± 0.02 a	1.02 ± 0.02 a	1.02 ± 0.02 a	1.05 ± 0.01 a	0.96 ± 0.03 a	1.01 ± 0.03 a	1.07 ± 0.02 a	3.5	Low
Dl	1.54 ± 0.01 a	1.53 ± 0.03 a	1.59 ± 0.09 a	1.49 ± 0.01 a	1.46 ± 0.05 a	1.66 ± 0.08 a	1.57 ± 0.06 a	1.73 ± 0.09 a	5.6	Medium
Dup	0.76 ± 0.02 a	0.80 ± 0.02 a	0.78 ± 0.04 a	0.76 ± 0.0 a	0.73 ± 0.02 a	0.85 ± 0.04 a	0.81 ± 0.02 a	0.86 ± 0.08 a	5.3	Medium
Dm	1.19 ± 0.03 a	1.17 ± 0.02 a	1.22 ± 0.05 a	1.21 ± 0.02 a	1.19 ± 0.05 a	1.30 ± 0.02 a	1.25 ± 0.04 a	1.33 ± 0.13 a	4.6	Low
mycl	640 ± 20 a	649 ± 10 a	628 ± 33 a	614 ± 15 a	635 ± 16 a	581 ± 18 a	623 ± 26 a	620 ± 58 a	3.3	Low
sbeg	0.77 ± 0.01 a	0.74 ± 0.01 a	0.80 ± 0.06 a	0.73 ± 0.01 a	0.73 ± 0.04 a	0.81 ± 0.04 a	0.76 ± 0.05 a	0.88 ± 0.02 a	6.5	Medium
n1Br	2.9 ± 0.2 a	2.9 ± 0.2 a	3.0 ± 0.2 a	3.0 ± 0.0 a	2.7 ± 0.3 a	3.3 ± 0.3 a	3.3 ± 0.2 a	3.0 ± 0.3 a	7.0	Medium
nBrO	2.2 ± 0.1 a	2.3 ± 0.1 a	2.2 ± 0.1 a	2.1 ± 0.0 a	2.1 ± 0.1 a	2.4 ± 0.1 a	2.4 ± 0.2 a	2.1 ± 0.1 a	5.3	Medium
nBol	4.0 ± 0.2 a	4.2 ± 0.4 a	4.3 ± 0.5 a	4.0 ± 0.1 a	3.7 ± 0.3 a	4.9 ± 0.6 a	4.7 ± 0.4 a	4.0 ± 0.4 a	9.4	Medium
StPr	953 ± 31 bc	840 ± 35 ab	847 ± 41 ab	833 ± 19 ab	837 ± 18 ab	**767 ± 43 a**	870 ± 52 abc	1023 ± 27 c	9.2	Medium
LFPr	100.4 ± 2.4 ab	98.2 ± 4.6 ab	92.3 ± 14.9 a	92.6 ± 9.7 a	98.2 ± 4.7 ab	80.3 ± 6.0 a	95.9 ± 2.5 ab	136.4 ± 12 b	16.4	High
LF%	10.6 ± 0.1 a	11.7 ± 0.4 a	10.8 ± 1.3 a	11.1 ± 0.9 a	11.7 ± 0.3 a	10.5 ± 0.3 a	11.1 ± 0.4 a	13.3 ± 1.2 a	8.2	Medium
SePr	319 ± 18 b	261 ± 21 ab	**270 ± 1 a**	269 ± 7 ab	267 ± 9 ab	**258 ± 9 a**	284 ± 10 ab	272 ± 3 ab	7.1	Medium
Se1000	5.0 ± 0.1 a	5.0 ± 0.0 a	5.0 ± 0.0 a	5.0 ± 0.1 a	5.1 ± 0.0 a	5.0 ± 0.1 a	5.0 ± 0.0 a	**5.6 ± 0.0 b**	4.1	Low
Str	27.7 ± 0.2 a	27.6 ± 0.7 a	27.5 ± 0.9 a	26.5 ± 0.8 a	27.8 ± 0.3 a	27.1 ± 1.0 a	25.5 ± 0.5 a	28.5 ± 1.1 a	3.4	Low
Flex	61.5 ± 2.3 b	56.3 ± 2.2 ab	62.0 ± 1.0 b	57.3 ± 2.6 ab	58.0 ± 2.1 ab	56.0 ± 2.0 ab	55 ± 1.7 ab	48.7 ± 3.5 a	7.3	Medium
Fin	268 ± 16 a	267 ± 17 a	252 ± 42 a	255 ± 44 a	274 ± 31 a	213 ± 19 a	239 ± 19 a	199 ± 6 a	11.1	High
Qo	19.8 ± 0.2 b	19.0 ± 0.0 ab	19.7 ± 0.3 b	19.0 ± 0.0 ab	19.3 ± 0.3 ab	19.3 ± 0.3 ab	19.0 ± 0.0 ab	**18.3 ± 0.3 a**	2.4	Low
Qc	17.3 ± 0.4 a	16.7 ± 0.4 a	17.1 ± 0.8 a	16.4 ± 0.7 a	17.0 ± 0.3 a	15.9 ± 0.5 a	15.8 ± 0.1 a	15.3 ± 0.3 a	4.4	Low
sp1	15.7 ± 0.5 a	13.0 ± 0.4 a	15.5 ± 0.4 a	16.4 ± 0.8 ab	15.6 ± 1.1 a	15.5 ± 1.8 a	15.0 ± 0.5 a	**20.4 ± 0.9 b**	13.1	High
sp2	6.9 ± 0.1 a	6.9 ± 0.1 a	7.7 ± 0.4 a	7.1 ± 0.4 a	7.3 ± 0.4 a	7.3 ± 0.5 a	7.6 ± 0.2 a	**10.1 ± 0.4 b**	13.5	High
sps	22.6 ± 0.5 a	19.9 ± 0.3 a	23.1 ± 0.4 a	23.5 ± 1.1 a	22.9 ± 1.5 a	22.8 ± 2.1 a	22.6 ± 0.6 a	**30.4 ± 1.3 b**	12.8	High

^1^ Plant traits are described in Section 4. Average ± standard error. Results, which are marked by the same letter, are not statistically different (One-Way ANOVA, Tukey, *p* > 0.05). ^2^ Storage of seeds for 2 months at room temperature (cont), −10 °C (m10), −30 °C (m30), −50 °C (m50), −80 °C (m80) and in liquid nitrogen (N) or storage for 24 years at −10 °C (LT). N-stored seeds were plunged directly into liquid nitrogen (Nd) or lowered in temperature gradually to −70 °C before immersion in liquid nitrogen (Ng). ^3^ Overall coefficient of variation = (Standard deviation/Average value) * 100. To calculate this coefficient, average values of the eight treatments compared were considered. The higher the difference between the eight compared average values, the higher the overall coefficient of variation (according to [120]). ^4^ Min OCV recorded = 1.1%; Max OCV recorded = 16.4%; Max−Min = 15.3%; (Max−Min)/3 = 5.1%. Classification of the OCVs: “low”—from 1.1 to 5.1%; “medium”—from 5.1 to 10.2%; and “high”—from 10.2 to 16.4% (according to [120]). ^5^ Values other than the control are shown in bold.

**Table 2:** Variation in characteristics of flax plants grown in field trials after different types of seed storage in the second year of the experiment (sowing with reproduced seeds).

Trait ^1^	Cont ^2^	m10	m30	m50	m80	Ng	Nd	LT	OCV, % ^3^	OCV, Level ^4^
germ	85.2 ± 1.5 a	84.1 ± 1.4 a	85.9 ± 0.1 a	85.0 ± 2.3 a	87.0 ± 1.1 a	82.3 ± 1.0 a	81.1 ± 0.2 a	85.8 ± 1.1 a	2.3	Low
g-f	37.0 ± 0.5 a	36.3 ± 1.2 a	37.3 ± 0.3 a	37.0 ± 0.0 a	37.0 ± 0.0 a	36.7 ± 2.2 a	36.7 ± 1.2 a	37.7 ± 0.3 a	1.1	Low
f-m	23.0 ± 1.1 a	24.0 ± 1.0 a	24.3 ± 1.2 a	22.3 ± 0.3 a	24.0 ± 0.6 a	25.0 ± 1.5 a	25.7 ± 0.7 a	23.3 ± 0.9 a	4.5	Low
g-m	60.0 ± 0.8 a	60.3 ± 1.3 a	61.7 ± 1.3 a	59.3 ± 0.3 a	61.0 ± 0.6 a	61.7 ± 0.9 a	62.3 ± 0.9 a	61.0 ± 1.0 a	1.6	Low
Hp	78.1 ± 1.5 a	75.3 ± 1.3 a	74.6 ± 0.9 a	75.1 ± 1.9 a	81 ± 1.3 a	75.9 ± 1.7 a	75.8 ± 3.9 a	77.5 ± 3.8 a	2.8	Low
Hs	62.7 ± 1.6 a	61.1 ± 1.4 a	60.2 ± 0.7 a	62.5 ± 1.4 a	65.7 ± 1.1 a	58.1 ± 2.4 a	59.9 ± 2.9 a	66.9 ± 3.8 a	4.8	Low
Hb	72.2 ± 1.3 a	69.8 ± 0.5 a	69.2 ± 0.9 a	70.0 ± 1.8 a	75.2 ± 1.3 a	69.3 ± 1.7 a	69.2 ± 3.1 a	73.5 ± 3.7 a	3.2	Low
Hinf	15.4 ± 1.2 ab	14.2 ± 2.7 ab	14.4 ± 1.0 ab	12.6 ± 1 ab	15.3 ± 0.4 ab	17.8 ± 1.4 b	15.8 ± 2 ab	10.6 ± 0.5 a	15.0	Medium
nL	75.9 ± 2.1 a	66.2 ± 5.4 a	69.6 ± 1.6 a	75.0 ± 2.0 a	78.1 ± 0.4 a	72.9 ± 6.7 a	71.0 ± 0.9 a	78.2 ± 1.3 a	5.8	Low
INode	0.83 ± 0.0 a	0.93 ± 0.06 a	0.87 ± 0.03 a	0.83 ± 0.03 a	0.84 ± 0.02 a	0.81 ± 0.08 a	0.84 ± 0.03 a	0.86 ± 0.05 a	4.3	Low
Dl	2.05 ± 0.06 a	2.01 ± 0.09 a	1.84 ± 0.07 a	1.88 ± 0.08 a	2.06 ± 0.01 a	2.07 ± 0.05 a	2.05 ± 0.13 a	1.88 ± 0.05 a	4.8	Low
Dup	1.57 ± 0.05 a	1.55 ± 0.1 a	1.42 ± 0.04 a	1.47 ± 0.03 a	1.53 ± 0.05 a	1.53 ± 0.01 a	1.57 ± 0.07 a	1.37 ± 0.04 a	4.9	Low
Dm	1.11 ± 0.06 a	1.07 ± 0.04 a	1.02 ± 0.04 a	0.99 ± 0.07 a	1.05 ± 0.01 a	1.16 ± 0.02 a	1.12 ± 0.1 a	0.92 ± 0.05 a	7.4	Low
mycl	401 ± 23 ab	396 ± 17 ab	423 ± 6 ab	426 ± 18 ab	431 ± 4 ab	380 ± 20 a	384 ± 25 ab	492 ± 43 b	8.6	Medium
sbeg	0.94 ± 0.01 a	0.94 ± 0.09 a	0.82 ± 0.08 a	0.89 ± 0.04 a	1.01 ± 0.04 a	0.91 ± 0.04 a	0.92 ± 0.07 a	0.95 ± 0.02 a	5.8	Low
n1Br	4.3 ± 0.0 a	3.9 ± 0.3 a	4.0 ± 0.1 a	3.9 ± 0.0 a	4.2 ± 0.1 a	4.5 ± 0.3 a	4.3 ± 0.2 a	3.9 ± 0.2 a	5.7	Low
nBrO	2.3 ± 0.1 a	2.1 ± 0.1 a	2.1 ± 0.1 a	2.1 ± 0.1 a	2.2 ± 0.1 a	2.3 ± 0.1 a	2.4 ± 0.2 a	1.9 ± 0.2 a	8.1	Medium
nBol	11.5 ± 0.7 ab	11.2 ± 1.9 ab	10.2 ± 0.7 ab	10.0 ± 0.7 ab	10.6 ± 0.5 ab	12.9 ± 0.4 b	12.0 ± 1.4 ab	7.8 ± 0.7 a	14.5	Medium
StPr	558 ± 29 a	488 ± 14 a	457 ± 15 a	528 ± 47 a	690 ± 93 a	527 ± 59 a	462 ± 42 a	452 ± 56 a	15.2	Medium
LFPr	55.8 ± 4.2 a	50.0 ± 2.9 a	50.0 ± 2.9 a	73.3 ± 7.3 a	86.7 ± 16.9 a	51.7 ± 10.1 a	51.7 ± 4.4 a	65.0 ± 10.4 a	22.2	High
LF%	10.0 ± 0.5 a	10.2 ± 0.3 a	10.9 ± 0.6 ab	**13.9 ± 0.6 bc ^5^**	12.3 ± 0.7 abc	9.6 ± 0.8 a	11.2 ± 0.1 ab	**14.3 ± 0.9 c**	15.4	Medium
SePr	140 ± 9 ab	125 ± 10 ab	121 ± 5 ab	137 ± 12 ab	205 ± 33 b	155 ± 31 ab	128 ± 13 ab	87 ± 11 a	24.5	High
Se1000	3.5 ± 0.0 ab	3.6 ± 0.1 ab	3.6 ± 0.0 ab	3.6 ± 0.1 ab	3.8 ± 0.1 ab	3.5 ± 0.1 a	3.6 ± 0.0 ab	4.0 ± 0.1 b	4.6	Low
Str	21.2 ± 1.5 ab	21.2 ± 0.8 ab	22.6 ± 0.9 ab	22.3 ± 0.4 ab	24.8 ± 1.2 b	21.0 ± 2.0 ab	18.1 ± 1.8 a	25.5 ± 1.2 b	10.6	Medium
Flex	57.8 ± 1.9 a	61.7 ± 6.0 a	63.7 ± 5.8 a	63.3 ± 1.5 a	61.7 ± 3.8 a	59.3 ± 3.4 a	55.3 ± 1.5 a	54.0 ± 3.2 a	6.1	Low
Fin	179 ± 13 a	196 ± 13 a	202 ± 4 a	198 ± 10 a	174 ± 30 a	218 ± 31 a	199 ± 52 a	212 ± 42 a	7.5	Low
Qo	19.0 ± 0.5 a	19.7 ± 0.3 a	20.0 ± 0.6 a	20.0 ± 0.6 a	20.3 ± 0.7 a	19.0 ± 0.6 a	18.0 ± 0.6 a	19.3 ± 0.3 a	3.9	Low
Qc	14.4 ± 0.4 a	15.1 ± 0.6 a	15.6 ± 0.4 a	15.5 ± 0.1 a	15.5 ± 0.8 a	15.1 ± 1.1 a	13.8 ± 1.0 a	15.4 ± 0.5 a	4.1	Low

^1^ Plant traits are as described in Section 4. Averages ± standard errors are reported. Results marked by the same letter are not statistically different (One-way ANOVA, Tukey, *p* > 0.05). ^2^ Reproduction of seeds after storage for 2 months at room temperature (cont), −10 °C (m10), −30 °C (m30), −50 °C (m50), −80 °C (m80) and in liquid nitrogen (N) or storage for 24 years at −10 °C (LT). N-stored seeds were plunged directly into liquid nitrogen (Nd) or lowered in temperature gradually to −70 °C before immersion in liquid nitrogen (Ng). ^3^ Overall coefficient of variation = (Standard deviation/Average) × 100. To calculate this coefficient, average values of the characters after eight treatments were compared. The greater the difference between the eight compared average values, the higher the overall coefficient of variation (according to [120]). ^4^ Min OCV recorded = 1.1%; Max OCV recorded = 24.5%; Max−Min = 23.4%; (Max−Min)/3 = 7.8%. Classification of the OCVs: “low” from 1.1 to 7.8%; “medium” from 7.8 to 15.6%; and “high” from 15.6 to 24.5% (according to [120]). ^5^ Values other than the control are shown **in bold**.

**Table 3:** Description and abbreviations of evaluated flax characters.

Abbreviation	Characters	Material Sampling
	**Germination**	
germL	Laboratory seeds germination ability after storage, %	100 seeds
germ	Field germination ability, %	All plants from the plot
	**Vegetation period**	
g-f	Period germination—flowering, days	Plot
f-m	Period flowering—maturity, days	Plot
g-m	Period germination—maturity, days	Plot
	**Morphological characters**	
Hp	Total plant height, cm	20 plants
Hs	Plant height from cotyledons to inflorescence, cm	20 plants
Hb	Plant height from cotyledons to the first boll, cm	20 plants
Hinf	Inflorescence length, cm	20 plants
nL	Number of leaves on the stem	20 plants
INode	Average length of internodes, cm	20 plants
Dl	Low-stem diameter, mm	20 plants
Dup	Upper-stem diameter, mm	20 plants
Dm	Middle-stem diameter, mm	20 plants
mycl	Ratio Hs/Dm	20 plants
sbeg	Difference between low- and upper-stem diameter (Dl-Dup), mm	20 plants
n1Br	Number of the main branches in inflorescence	20 plants
nBrO	Number of inflorescence branching orders	20 plants
nBol	Number of bolls	20 plants
	**Productivity**	
StPr	Straw production, g/m^2^	
LFPr	Long-fiber production after water retting, g/m^2^	Plot
%LF	% of long technical fiber after water retting, %	Plot
SePr	Seeds production, g/m^2^	Plot
Se1000	Weight of 1000 seeds, g	Plot
	**Fiber quality**	
Str	Strength of long technical fiber, N	15 measurements/Plot
Flex	Flexibility of long technical fiber, mm	15 measurements/Plot
Fin	Fineness of long technical fiber, m/g	3 measurements/Plot
Qo	Quality number of long technical fiber, estimated organoleptically	Plot
Qc	Calculated quality number of long technical fiber (0.2 × **Str** + 0.1 × **Flex** + 0.013 × **Fin** + 2.1)	Plot
	**Snap point (only in 2020)**	
sp1	Length from cotyledon to snap point, cm	5 plants
sp2	Length from stem apex to snap point, cm	5 plants
spS	Length from cotyledon to apex, cm	5 plants

## Data Availability

All data is presented in the article.

## Supplementary Materials

The following supporting information can be downloaded at: https://www.mdpi.com/article/10.3390/plants15040602/s1, **Table S1:** Probable similarity between characters of plants, grown from seeds after different types of storage, and control seeds in the first year after seeds treatment; **Table S2:** Probable similarity between characters of plants grown from seeds after different types of seeds’ storage and plants grown from control seeds (sowing with reproduced seeds); **Table S3:** Two-way ANOVA analyses of storage type, cycle of seeds reproduction and their interaction influence on the manifestation of morphological, phenological and economically valuable characters. Post hoc analysis LSD (Fisher) and HSD (Tukey) difference between different types of storages in 2020 and 2021 years (reduced average values of characters in 2020 and 2021); **Table S4:** Modification of flax plants characters after long-term seeds storage in year of removing seeds from the storage and in the next year after reproduction. Probability of similarity between plants, grown from seeds after long-term storage and control seeds in the first and second (sowing with reproduced seeds) year after seeds.
