# Supplementary material for: The Influence of Cryopreservation and Low-Temperature Seed Storage on the Morphological and Agronomical Characteristics of Fiber Flax
Source: Plants (Basel). 2026 Feb 13;15(4):602. doi: 10.3390/plants15040602 (PMC12944033; doi:10.3390/plants15040602)
Supplement: Supplementary file 1 [file plants-15-00602-s001.zip › Pavlov25_Table S1_pl2.pdf]

**Table S1.** Probable similarity between characters of plants, grown from seeds after different types of storage, and control seeds in the first year after seeds treatment

| | m10 | | | m30 | | | m50 | | | m80 | | | Ng | | | Nd | | | LT | | |
|---|---|---|---|---|---|---|---|---|---|---|---|---|---|---|---|---|---|---|---|---|---|
| **Trait** | **T-st[1]** | **M-W** | **HSD** | **T-st** | **M-W** | **HSD** | **T-st** | **M-W** | **HSD** | **T-st** | **M-W** | **HSD** | **T-st** | **M-W** | **HSD** | **T-st** | **M-W** | **HSD** | **T-st** | **M-W** | **HSD** |
| germ[2] | 0.28 | 0.51 | 0.89 | 0.36 | 0.51 | 0.95 | 0.07 | **0.05*** | 0.67 | 0.10 | **0.05*** | 0.92 | **0.02*** | **0.0495*** | 0.0537 | **0.01*** | **0.0495*** | 0.43 | 0.06 | **0.05*** | 0.97 |
| g-f | **0.03*** | **0.04*** | 0.10 | 0.49 | 0.49 | 0.96 | 1.00 | 1.00 | 1.00 | 1.00 | 1.00 | 1.00 | 0.49 | 0.49 | 0.96 | 1.00 | 1.00 | 1.00 | **0.00*** | **0.05*** | **0.00*** |
| f-m | 0.49 | 0.49 | 1.00 | 0.49 | 0.49 | 1.00 | 0.41 | 0.49 | 0.99 | 1.00 | 1.00 | 1.00 | 0.39 | 0.49 | 0.90 | 0.41 | 0.49 | 0.99 | 1.00 | 1.00 | 1.00 |
| g-m | 0.21 | 0.18 | 0.98 | 0.21 | 0.18 | 0.98 | 0.41 | 0.49 | 0.98 | 1.00 | 1.00 | 1.00 | 0.22 | 0.18 | 0.60 | 0.41 | 0.49 | 0.98 | **0.00*** | **0.05*** | **0.03*** |
| Hp | 0.56 | 0.51 | 1.00 | 0.54 | 0.51 | 1.00 | 0.58 | 0.51 | 0.99 | 0.57 | 0.51 | 1.00 | 0.71 | 0.83 | 1.00 | **0.04*** | **0.05*** | 0.61 | **0.05*** | **0.05*** | 0.18 |
| Hs | 0.95 | 0.83 | 1.00 | 0.90 | 0.83 | 1.00 | 0.50 | 0.51 | 0.98 | 0.62 | 0.51 | 1.00 | 0.71 | 0.83 | 1.00 | 0.20 | 0.18 | 0.95 | **0.02*** | **0.05*** | 0.10 |
| Hb | 0.62 | 0.51 | 1.00 | 0.75 | 0.51 | 1.00 | 0.65 | 0.83 | 1.00 | 0.66 | 0.83 | 1.00 | 0.91 | 0.83 | 1.00 | **0.04*** | **0.05*** | 0.66 | **0.03*** | **0.05*** | 0.09 |
| Hinf | 0.08 | **0.05*** | 0.92 | 0.51 | 0.28 | 1.00 | 0.36 | 0.38 | 1.00 | 0.61 | 0.66 | 1.00 | 0.25 | 0.28 | 0.76 | 0.22 | 0.27 | 0.76 | 0.40 | 0.28 | 0.98 |
| nL | 0.49 | 0.83 | 0.98 | 0.74 | 0.51 | 1.00 | 0.30 | 0.28 | 0.95 | 0.22 | 0.13 | 0.81 | 0.23 | 0.28 | 0.97 | 0.63 | 0.83 | 1.00 | 0.95 | 0.83 | 1.00 |
| INode | 0.34 | 0.28 | 0.97 | 0.68 | 0.83 | 1.00 | 0.55 | 0.51 | 1.00 | 0.11 | **0.05*** | 0.90 | 0.23 | 0.28 | 0.88 | 0.96 | 0.51 | 1.00 | 0.08 | 0.13 | 0.44 |
| Dl | 0.95 | 0.83 | 1.00 | 0.62 | 0.83 | 1.00 | 0.06 | 0.13 | 1.00 | 0.21 | 0.51 | 0.99 | 0.20 | 0.28 | 0.84 | 0.58 | 0.83 | 1.00 | 0.09 | **0.05*** | 0.36 |
| Dup | 0.34 | 0.83 | 1.00 | 0.66 | 0.83 | 1.00 | 0.99 | 0.51 | 1.00 | 0.36 | 0.28 | 1.00 | 0.15 | 0.28 | 0.79 | 0.19 | 0.28 | 0.98 | 0.35 | 0.28 | 0.69 |
| Dm | 0.57 | 0.51 | 1.00 | 0.67 | 0.83 | 1.00 | 0.56 | 0.83 | 1.00 | 0.92 | 0.83 | 1.00 | **0.05*** | **0.05*** | 0.87 | 0.32 | 0.28 | 0.99 | 0.35 | 0.51 | 0.65 |
| mycl | 0.67 | 0.83 | 1.00 | 0.78 | 0.83 | 1.00 | 0.38 | 0.28 | 1.00 | 0.90 | 0.83 | 1.00 | 0.10 | 0.13 | 0.82 | 0.65 | 0.51 | 1.00 | 0.77 | 0.51 | 1.00 |
| sbeg | 0.10 | **0.05*** | 0.99 | 0.62 | 0.83 | 1.00 | **0.02*** | **0.05*** | 0.99 | 0.35 | 0.51 | 0.98 | 0.34 | 0.51 | 0.99 | 0.84 | 0.51 | 1.00 | **0.01*** | **0.05*** | 0.41 |
| n1Br | 0.90 | 0.83 | 1.00 | 0.74 | 0.38 | 1.00 | 0.58 | 0.51 | 1.00 | 0.65 | 0.51 | 1.00 | 0.25 | 0.13 | 0.83 | 0.16 | 0.27 | 0.83 | 0.72 | 0.83 | 1.00 |
| nBrO | 0.33 | 0.26 | 0.99 | 0.85 | 0.82 | 1.00 | 0.25 | 0.27 | 1.00 | 0.29 | 0.38 | 0.99 | 0.29 | 0.28 | 0.90 | 0.40 | 0.38 | 0.90 | 0.53 | 0.51 | 1.00 |
| nBol | 0.62 | 0.66 | 1.00 | 0.59 | 0.66 | 1.00 | 0.98 | 0.51 | 1.00 | 0.50 | 0.28 | 1.00 | 0.23 | 0.13 | 0.75 | 0.19 | 0.28 | 0.86 | 0.96 | 0.83 | 1.00 |
| StPr | 0.07 | **0.05*** | 0.36 | 0.11 | **0.05*** | 0.42 | **0.03*** | **0.05*** | 0.29 | **0.03*** | **0.05*** | 0.32 | **0.02*** | **0.05*** | **0.03*** | 0.24 | 0.27 | 0.70 | 0.17 | 0.12 | 0.84 |
| LFPr | 0.69 | 0.51 | 1.00 | 0.62 | 0.83 | 1.00 | 0.48 | 0.51 | 1.00 | 0.69 | 0.51 | 1.00 | **0.04*** | **0.05*** | 0.68 | 0.26 | 0.28 | 1.00 | **0.04*** | **0.05*** | 0.11 |
| LF% | 0.07 | **0.05*** | 0.97 | 0.92 | 0.51 | 1.00 | 0.67 | 0.51 | 1.00 | **0.03*** | **0.05*** | 0.96 | 0.64 | 0.83 | 1.00 | 0.34 | 0.28 | 1.00 | 0.08 | **0.05*** | 0.25 |
| SePr | 0.11 | 0.13 | **0.05*** | 0.05 | **0.05*** | 0.12 | 0.06 | **0.05*** | 0.12 | 0.07 | **0.05*** | 0.09 | **0.04*** | **0.05*** | **0.03*** | 0.17 | 0.13 | 0.44 | 0.06 | 0.05 | 0.15 |
| Se1000 | 1.00 | 0.82 | 1.00 | 1.00 | 0.82 | 1.00 | 1.00 | 1.00 | 1.00 | 0.70 | 0.82 | 1.00 | 0.59 | 0.51 | 0.99 | 0.45 | 0.37 | 0.99 | **0.00*** | **0.04*** | **0.00*** |
| Str | 0.87 | 0.83 | 1.00 | 0.87 | 0.83 | 1.00 | 0.20 | 0.08 | 0.94 | 0.79 | 0.66 | 1.00 | 0.61 | 0.51 | 1.00 | **0.01*** | **0.05*** | 0.48 | 0.51 | 0.83 | 0.99 |
| Flex | 0.18 | 0.28 | 0.74 | 0.85 | 0.82 | 1.00 | 0.30 | 0.28 | 0.89 | 0.32 | 0.28 | 0.95 | 0.15 | 0.12 | 0.68 | 0.09 | 0.13 | 0.50 | **0.04*** | **0.05*** | **0.02*** |
| Fin | 0.99 | 0.83 | 1.00 | 0.75 | 0.83 | 1.00 | 0.80 | 0.51 | 1.00 | 0.85 | 0.83 | 1.00 | 0.09 | 0.13 | 0.84 | 0.31 | 0.28 | 0.99 | **0.02*** | **0.05*** | 0.64 |
| Qo | **0.01*** | **0.03*** | 0.29 | 0.68 | 0.80 | 1.00 | **0.01*** | **0.03*** | 0.29 | 0.25 | 0.24 | 0.82 | 0.25 | 0.24 | 0.82 | **0.01*** | **0.03*** | 0.29 | **0.02*** | **0.04*** | **0.01*** |
| Qc | 0.39 | 0.51 | 0.99 | 0.84 | 0.83 | 1.00 | 0.35 | 0.28 | 0.91 | 0.64 | 0.83 | 1.00 | 0.11 | 0.13 | 0.49 | **0.02*** | **0.05*** | 0.41 | **0.01*** | **0.05*** | 0.12 |
| sp1 | **0.01*** | **0.05*** | 0.43 | 0.71 | 0.83 | 1.00 | 0.49 | 0.51 | 1.00 | 0.91 | 0.83 | 1.00 | 0.90 | 0.51 | 1.00 | 0.38 | 0.28 | 1.00 | **0.01*** | **0.05*** | **0.04*** |
| sp2 | 0.82 | 0.83 | 1.00 | 0.17 | 0.28 | 0.71 | 0.64 | 0.83 | 1.00 | 0.34 | 0.51 | 0.98 | 0.45 | 0.51 | 0.99 | **0.05*** | **0.05*** | 0.83 | **0.00*** | **0.05*** | **0.00*** |
| spS | **0.01*** | **0.05*** | 0.70 | 0.44 | 0.28 | 1.00 | 0.51 | 0.51 | 1.00 | 0.88 | 0.51 | 1.00 | 0.95 | 1.00 | 1.00 | 0.99 | 0.83 | 1.00 | **0.00*** | **0.05*** | **0.00*** |

[1] – Different types of seeds' storage: m10, m30, m50, m80 - freezers at -10°C, -30°C, -50°C, -80°C, respectively. Nd - direct immersion in liquid nitrogen; Ng - gradual freezing in liquid nitrogen.

[2] –T-st – Student's t-test, M-W – Mann-Whitney U test, HSD – Tukey's HSD (honestly significant difference) test.

[3] – germ - field germinating ability, %; g-f - period germination - flowering, days, f-m - period flowering - maturity, days; g-m - period germination - maturity, days; Hp - total plant height, cm; Hs - plant height from cotyledons to inflorescence, cm; Hb - plant height from cotyledons to the first boll, cm; Hinf - inflorescence length, cm; nL - number of leaves on the stem; INode - average length of internodes, cm; Dl - low stem diameter, mm; Dup - upper stem diameter, mm; Dm - middle stem diameter, mm; mycl - ratio Hs/Dm; sbeg - difference between low and and upper stem diameter (Dl-Dup), mm; n1Br - number of the main branches in inflorescence; nBrO - number of inflorescence branching orders; nBol - number of bolls; StPr - straw

production, g/m$^2$; LFPr - long fibre production after water retting, g/m$^2$; %LF - % of long technical fibre after water retting, %; SePr - seeds production, g/m2; Se1000 - weight of 1000 seeds, g; Flex - flexibility of long technical fibre, mm; Fin - fineness of long technical fibre, m/g; Qo - quality number of long technical fibre, estimated organoleptically ; Qc - calculated quality number of long technical fibre (0.2×Str+0.1×Flex + 0.013× Fin+2.1); sp1 - length from cotyledon to snap point, cm; sp2 - length from stem apex to snap point, cm; spS - length from cotyledon to apex, cm

* - Significant differences are marked in bold and asterisk.
