# Supplementary material for: The Influence of Cryopreservation and Low-Temperature Seed Storage on the Morphological and Agronomical Characteristics of Fiber Flax
Source: Plants (Basel). 2026 Feb 13;15(4):602. doi: 10.3390/plants15040602 (PMC12944033; doi:10.3390/plants15040602)
Supplement: Supplementary file 1 [file plants-15-00602-s001.zip › Pavlov25_Table S2_pl2.pdf]

Table S2. Probable similarity between characters of plants grown from seeds after different types of seeds' storage and plants grown from control seeds (sawing with reproduced seeds)

	m10[1]			m30			m50			m80			Ng			Nd			LT		
**Trait**	**T-st[2]**	**M-W**	**HSD**	**T-st**	**M-W**	**HSD**	**T-st**	**M-W**	**HSD**	**T-st**	**M-W**	**HSD**	**T-st**	**M-W**	**HSD**	**T-st**	**M-W**	**HSD**	**T-st**	**M-W**	**HSD**
germ[3]	0.62	0.51	1.00	0.68	0.51	1.00	0.95	0.83	1.00	0.38	0.28	0.97	0.17	0.28	0.72	0.05	**0.05***	0.36	0.77	0.83	1.00
g-f	0.64	0.82	1.00	0.61	0.82	1.00	1.00	0.48	1.00	1.00	0.48	1.00	0.89	0.51	1.00	0.81	0.65	1.00	0.33	0.26	1.00
f-m	0.53	0.27	0.99	0.45	0.28	0.97	0.57	0.51	1.00	0.45	0.51	0.99	0.34	0.28	0.82	0.10	**0.05***	0.54	0.82	0.83	1.00
g-m	0.84	0.82	1.00	0.34	0.27	0.91	0.47	0.51	1.00	0.36	0.38	0.99	0.23	0.28	0.91	0.12	0.08	0.66	0.47	0.82	0.99
Hp	0.24	0.28	0.99	0.12	0.13	0.95	0.29	0.28	0.98	0.24	0.28	0.99	0.39	0.51	1.00	0.60	0.83	1.00	0.88	0.51	1.00
Hs	0.48	0.83	1.00	0.21	0.28	0.99	0.93	0.83	1.00	0.20	0.28	0.97	0.18	0.28	0.78	0.44	0.83	0.98	0.37	0.51	0.85
Hb	0.14	0.10	0.99	0.12	**0.05***	0.96	0.39	0.28	0.99	0.18	0.13	0.96	0.24	0.28	0.96	0.42	0.51	0.96	0.76	0.51	1.00
Hinf	0.71	0.83	1.00	0.56	0.28	1.00	0.14	0.13	0.85	0.94	0.83	1.00	0.27	0.28	0.93	0.86	0.83	1.00	**0.02***	**0.05***	0.32
nL	0.17	0.28	0.46	0.07	**0.05***	0.86	0.76	0.51	1.00	0.36	0.51	1.00	0.68	0.51	1.00	0.09	**0.05***	0.96	0.40	0.28	1.00
INode	0.20	0.51	0.75	0.27	0.51	1.00	0.92	0.51	1.00	0.61	0.83	1.00	0.81	0.51	1.00	0.73	0.51	1.00	0.68	0.51	1.00
Dl	0.73	0.83	1.00	0.09	0.13	0.54	0.16	0.28	0.76	0.85	0.51	1.00	0.79	0.51	1.00	1.00	0.51	1.00	0.10	**0.05***	0.74
Dup	0.75	0.51	1.00	0.22	0.28	0.93	0.11	0.13	0.76	0.44	0.51	0.99	0.37	0.51	1.00	0.88	0.83	1.00	**0.04***	**0.05***	0.31
Dm	0.77	0.83	1.00	0.13	**0.05***	0.63	0.36	0.28	0.92	0.53	0.83	1.00	0.59	0.83	1.00	0.99	0.51	1.00	0.07	**0.05***	0.26
mycl	0.86	0.83	1.00	0.42	0.51	1.00	0.44	0.51	0.99	0.28	0.28	0.98	0.54	0.51	1.00	0.64	0.51	1.00	0.13	0.13	0.15
sbeg	0.99	0.51	1.00	0.21	0.51	0.79	0.40	0.51	1.00	0.20	0.13	0.98	0.49	0.51	1.00	0.86	0.83	1.00	0.55	0.83	1.00
n1Br	0.22	0.08	0.71	0.13	0.13	0.98	**0.00***	**0.05***	0.85	0.44	0.50	1.00	0.46	0.51	0.99	0.88	0.51	1.00	0.09	**0.05***	0.71
nBrO	0.32	0.13	0.93	0.29	0.28	0.93	0.18	0.28	0.86	0.63	0.27	1.00	0.96	0.83	1.00	0.69	0.82	1.00	0.07	**0.05***	0.14
nBol	0.88	0.51	1.00	0.23	0.28	0.98	0.21	0.28	0.96	0.33	0.28	1.00	0.13	0.28	0.96	0.75	0.51	1.00	**0.02***	**0.05***	0.22
StPr	0.10	0.13	0.97	**0.04***	**0.05***	0.84	0.63	0.83	1.00	0.25	0.13	0.60	0.67	0.83	1.00	0.13	0.13	0.87	0.17	0.13	0.81
LFPr	0.31	0.27	1.00	0.31	0.27	1.00	0.10	0.10	0.83	0.15	**0.05***	0.26	0.72	0.82	1.00	0.53	0.49	1.00	0.46	0.49	0.99
LF%	0.72	0.83	1.00	0.28	0.28	0.96	**0.01***	**0.05***	**0.01***	0.05	**0.05***	0.21	0.71	0.83	1.00	0.07	**0.05***	0.87	**0.01***	**0.05***	**0.00***
SePr	0.33	0.28	1.00	0.14	0.13	1.00	0.88	0.83	1.00	0.13	**0.05***	0.25	0.65	0.51	1.00	0.49	0.28	1.00	**0.02***	**0.05***	0.51
Se1000	0.35	0.37	1.00	0.23	0.37	1.00	0.75	0.65	1.00	0.08	**0.05***	0.48	0.66	0.65	1.00	0.22	0.36	1.00	**0.03***	**0.05***	0.08
Str	0.98	0.83	1.00	0.46	0.83	0.99	0.50	0.83	1.00	0.13	**0.05***	0.54	0.95	0.83	1.00	0.26	0.13	0.73	0.09	**0.05***	0.33
Flex	0.58	0.51	1.00	0.39	0.51	0.95	0.08	**0.05***	0.96	0.42	0.28	1.00	0.72	0.83	1.00	0.36	0.28	1.00	0.36	0.38	1.00
Fin	0.42	0.51	1.00	0.16	0.13	1.00	0.32	0.28	1.00	0.87	0.51	1.00	0.32	0.28	0.98	0.73	0.51	1.00	0.49	0.51	0.99
Qo	0.33	0.26	0.98	0.26	0.27	0.87	0.26	0.27	0.87	0.18	0.26	0.64	1.00	1.00	1.00	0.26	0.18	0.87	0.61	0.82	1.00
Qc	0.44	0.28	1.00	0.12	0.13	0.91	0.06	**0.05***	0.95	0.30	0.28	0.95	0.62	0.51	1.00	0.60	0.83	1.00	0.20	0.28	0.97

[1] – Different types of seeds' storage: m10, m30, m50, m80 - freezers at -10°C, -30°C, -50°C, -80°C, respectively. Nd - direct immersion in liquid nitrogen; Ng - gradual freezing in liquid nitrogen.

[2] –T-st – Student's t-test, M-W – Mann-Whitney U test, HSD – Tukey's HSD (honestly significant difference) test.

[3] – germ - field germinating ability, %; g-f - period germination - flowering, days, f-m - period flowering - maturity, days; g-m - period germination - maturity, days; Hp - total plant height, cm; Hs - plant height from cotyledons to inflorescence, cm; Hb - plant height from cotyledons to the first boll, cm; Hinf - inflorescence length, cm; nL - number of leaves on the stem; INode - average length of internodes, cm; Dl - low stem diameter, mm; Dup - upper stem diameter, mm; Dm - middle stem diameter, mm; mycl - ratio Hs/Dm; sbeg - difference between low and and upper stem diameter (Dl-Dup), mm; n1Br - number of the main branches in inflorescence; nBrO - number of inflorescence branching orders; nBol - number of bolls; StPr - straw production, g/m2; LFPr - long fibre production after water retting, g/m2; %LF - % of long technical fibre after water retting, %; SePr - seeds production, g/m2; Se1000 - weight of 1000 seeds, g; Flex - flexibility of long technical fibre, mm;

Fin - fineness of long technical fibre, m/g; Qo - quality number of long technical fibre, estimated organoleptically ; Qc - calculated quality number of long technical fibre (0.2×Str+0.1×Flex + 0.013× Fin+2.1).

* - Significant differences are marked in bold and asterisk.
