# Supplementary material for: The Influence of Cryopreservation and Low-Temperature Seed Storage on the Morphological and Agronomical Characteristics of Fiber Flax
Source: Plants (Basel). 2026 Feb 13;15(4):602. doi: 10.3390/plants15040602 (PMC12944033; doi:10.3390/plants15040602)
Supplement: Supplementary file 1 [file plants-15-00602-s001.zip › Pavlov25_Table S4_pl2.pdf]

Table S4. Modification of flax plants characters after long term seeds storage in year of removing seeds from the storage and in the next year after reproduction. Probability of similarity between plants, grown from seeds after long terms storage and control seeds in the first and second (sawing with reproduced seeds) year after seeds treatment

Trait	First year after storage						Second year after storage					
	cont[1]	LT	T-st[2]	M-W	HSD	Influence[3]	cont	LT	T-st	M-W	HSD	Influence
germ	81.9±0.1a	75.5±2.4a	0.06	**0.05***	0.97		85.2±1.5a	85.8±1.1a	0.77	0.83	1.00	
g-f	37.0±0.3bc	34.3±0.3a	**0.00***	**0.05***	**0.00***	negative	37.0±0.5a	37.7±0.3a	0.33	0.26	1.00	
f-m	43.0±0.3a	43.0±0.6a	1.00	1.00	1.00		23.0±1.1a	23.3±0.9a	0.82	0.83	1.00	
g-m	80.0±0.3b	77.3±0.3a	**0.00***	**0.05***	**0.03***	negative	60.0±0.8a	61.0±1.0a	0.47	0.82	0.99	
Hp	85.7±0.9ab	90.0±1.4b	**0.05***	**0.05***	0.18	positive	78.1±1.5a	77.5±3.8a	0.88	0.51	1.00	
Hs	76.0±1.0ab	81.0±0.9b	**0.02***	**0.05***	0.10	positive	62.7±1.6a	66.9±3.8a	0.37	0.51	0.85	
Hb	82.2±0.9ab	87.4±1.2b	**0.03***	**0.05***	0.09	positive	72.2±1.3a	73.5±3.7a	0.76	0.51	1.00	
Hinf	9.7±0.3a	9.0±0.6a	0.40	0.28	0.98		15.4±1.2ab	10.6±0.5a	**0.02***	**0.05***	0.32	mb negative
nL	75.6±1.6a	75.5±1.0a	0.95	0.83	1.00		75.9±2.1a	78.2±1.3a	0.40	0.28	1.00	
INode	1.01±0.02a	1.07±0.02a	0.08	0.13	0.44		0.83±0.0a	0.86±0.05a	0.68	0.51	1.00	
Dl	1.54±0.01a	1.73±0.09a	0.09	**0.05***	0.36	mb positive	2.05±0.06a	1.88±0.05a	0.10	**0.05***	0.74	
Dup	0.76±0.02a	0.86±0.08a	0.35	0.28	0.69		1.57±0.05a	1.37±0.04a	**0.04***	**0.05***	0.31	mb negative
Dm	1.19±0.03a	1.33±0.13a	0.35	0.51	0.65		1.11±0.06a	0.92±0.05a	0.07	**0.05***	0.26	
mycl	640±20a	620±58a	0.77	0.51	1.00		401±23ab	492±43b	0.13	0.13	0.15	
sbeg	0.77±0.01a	0.88±0.02a	**0.01***	**0.05***	0.41	mb positive	0.94±0.01a	0.95±0.02a	0.55	0.83	1.00	
n1Br	2.9±0.2a	3.0±0.3a	0.72	0.83	1.00		4.3±0.0a	3.9±0.2a	0.09	**0.05***	0.71	
nBrO	2.2±0.1a	2.1±0.1a	0.53	0.51	1.00		2.3±0.1a	1.9±0.2a	0.07	**0.05***	0.14	
nBol	4.0±0.2a	4.0±0.4a	0.96	0.83	1.00		11.5±0.7ab	7.8±0.7a	**0.02***	**0.05***	0.22	ns negative
StPr	953±31bc	1023±27c	0.17	0.12	0.84		558±29a	452±56a	0.17	0.13	0.81	
LFPr	100.4±2.4ab	136.4±12b	**0.04***	**0.05***	0.11	positive	55.8±4.2a	65.0±10.4a	0.46	0.49	0.99	
LF%	10.6±0.1a	13.3±1.2a	0.08	**0.05***	0.25	ns positive	10.0±0.5a	14.3±0.9c	**0.01***	**0.05***	**0.00***	positive
SePr	319±18b	272±3ab	0.06	0.05	0.15	ns negative	140±9ab	87±11a	**0.02***	**0.05***	0.51	negative
Se1000	5.0±0.1a	5.6±0.0b	**0.00***	**0.04***	**0.00***	positive	3.5±0.0ab	4.0±0.1b	**0.03***	**0.05***	0.08	positive
Str	27.7±0.2a	28.5±1.1a	0.51	0.83	0.99		21.2±1.5ab	25.5±1.2b	0.09	**0.05***	0.33	mb positive
Flex	61.5±2.3b	48.7±3.5a	**0.04***	**0.05***	**0.02***	negative	57.8±1.9a	54.0±3.2a	0.36	0.38	1.00	
Fin	268±16a	199±6a	**0.02***	**0.05***	0.64	mb negative	179±13a	212±42a	0.49	0.51	0.99	
Qo	19.8±0.2b	18.3±0.3a	**0.02***	**0.04***	**0.01***	negative	19.0±0.5a	19.3±0.3a	0.61	0.82	1.00	
Qc	17.3±0.4a	15.3±0.3a	**0.01***	**0.05***	0.12	mb negative	14.4±0.4a	15.4±0.5a	0.20	0.28	0.97	
sp1	15.7±0.5a	20.4±0.9b	**0.01***	**0.05***	**0.04***	positive						
sp2	6.9±0.1a	10.1±0.4b	**0.00***	**0.05***	**0.00***	positive						
sps	22.6±0.5a	30.4±1.3b	**0.00***	**0.05***	**0.00***	positive						

[1] Average ± standard error. Results marked by the same letter are not statistically different (One-way ANOVA, Tukey, p>0.05).

[2] – T-st – Student's t-test, M-W – Mann-Whitney U test, HSD – Tukey's HSD (honestly significant difference) test.

* - Significant differences are marked in bold and asterisk.

[3] – mb – may be, ns – not significant
